# ﻿A new distinctive lineage of *Helix* (Gastropoda, Stylommatophora, Helicidae), with a guide to *Helix* species from mainland Greece

**DOI:** 10.3897/zookeys.1249.143635

**Published:** 2025-08-18

**Authors:** Ondřej Korábek, Petr Dolejš, Radovan Coufal, Lucie Juřičková, Kateřina Kubíková, Bernhard Hausdorf

**Affiliations:** 1 Department of Zoology, Faculty of Science, Charles University, Praha, Czech Republic Charles University Praha Czech Republic; 2 Department of Zoology, National Museum of the Czech Republic – Natural History Museum, Praha, Czech Republic National Museum of the Czech Republic – Natural History Museum Praha Czech Republic; 3 Department of Botany and Zoology, Faculty of Science, Masaryk University, Brno, Czech Republic Masaryk University Brno Czech Republic; 4 Leibniz Institute for the Analysis of Biodiversity Change, Zoological Museum, Hamburg, Germany Leibniz Institute for the Analysis of Biodiversity Change Hamburg Germany; 5 Universität Hamburg, Hamburg, Germany Universität Hamburg Hamburg Germany

**Keywords:** Balkans, distributions, diversity, endemics, land snail, phylogeny, species identification, taxonomy

## Abstract

Greece is home to numerous endemic land snail species, sometimes with highly restricted distributions. Several species of *Helix*, representing all three subgenera, live there. Although the genus was taxonomically revised in 2014, there remained some open questions and the distribution ranges of individual species are still incompletely known. The discovery of a new, narrowly endemic subspecies, *Helixpelagonesicathembones* Korábek, Juřičková & Hausdorf, **subsp. nov.**, is reported from the hills bordering the Thessalian Plain in the west. It was previously confused with *Helixschlaeflii*, but it was found that *H.schlaeflii* does not occur as far east as previously thought. Notably, the isolated populations from the tip of the Sithonia peninsula (Chalkidiki) that had previously been identified as *H.schlaeflii* also turned out to be related to *H.pelagonesica*. Although a divergent mitochondrial lineage was found there, morphological differences limited only to a different shell shape and lack of geographic separation led us to classify these populations as a form of *H.pelagonesicapelagonesica*. In addition, *Helixstraminea* is reported from Greece for the first time, with a population located near Kalambaka. All the *Helix* species from mainland Greece are illustrated and accompanied by descriptions to facilitate their recognition and maps of their distribution are provided.

## ﻿Introduction

The distribution of species diversity is uneven as the diversity generally increases towards the equator. Considering the distribution of European land snail diversity (Welter-Schultes 2012), we may recognize four largely latitudinal, geographically overlapping zones. The very north of Europe (taiga and tundra) is home to a depauperate fauna composed only of tiny species adapted to cold conditions that have very large distribution ranges (e.g. Schenková and Horsák 2013; Horsáková et al. 2019; Horsák et al. 2022). The temperate zone, at intermediate latitudes north of the Pyrenees, Alps and Carpathians, is home to often locally species-rich faunas consisting mostly of species that had to colonise these areas from glacial refugia and thus also often have rather large ranges (Hausdorf and Hennig 2003). Slightly further south is a zone of these glacial refugia, which already contains some endemic species (e.g. Cameron et al. 2016; Korábek et al. 2023b; Adamcová et al. 2024). The southernmost zone hosts, especially in the areas under the Mediterranean climatic regime, a high diversity of regional endemics (e.g. Cadevall and Orozco 2016; Cameron et al. 2023). The diversification there has a long history, resulting in not only endemic species, but even endemic genera (Welter-Schultes 2012). Furthermore, it is here in the south of Europe, where we may expect to find species with the smallest ranges, because of spatial heterogeneity and less severe extinctions due to a relatively lower impact of the glacial cycles. Indeed, some lineages differentiated on a very fine geographic scale (e.g. Mason et al. 2020).

The south of Europe consists of three peninsulas that differ in diversity and phylogenetic composition of their land snail faunas. The Balkans is the largest and most species-rich, followed by the Iberian Peninsula; the Apennine Peninsula has a relatively low diversity of land snails. In the Balkans, the diversity of land snails concentrates to the mountainous western part of the peninsula, culminating in an area roughly from Montenegro to Greece. Complex topography of the area and abundance of limestones created plenty of opportunities for the diversification of land snails, and the south of the Balkans, in particular Greece, also provided refugia allowing for the survival of relict lineages (as in Phaedusinae; Reischütz et al. 2016).

The diversity in Greece is high and only few groups have been investigated with genetic data. Therefore, there is a need for comprehensive taxonomic revisions employing modern genetic methods in many genera to clarify species limits. There may also still remain undescribed forms, if they have small ranges and/or cryptic lifestyle, and if they occur far from popular holiday destinations.

The diversity of the genus *Helix* Linnaeus, 1758 appears to be rather well documented. A morphology-based revision of the genus was published more than ten years ago (Neubert 2014), and Korábek et al. (2022) added an overview of the intraspecific mitochondrial diversity of most species. The exploration of the diversity of the genus in Greece would thus seem to be a done job. However, the sampling effort was geographically uneven and if there are extremely narrowly distributed lineages, they still might have escaped discovery, or at least a proper taxonomic treatment.

A new mitochondrial lineage of *Helix*, so divergent that it may represent an unknown species, has recently been recognized by sequencing of museum samples previously identified as *Helixschlaeflii* Mousson, 1859. One individual from western Thessaly yielded a sequence related to *Helixpelagonesica* (Rolle, 1898) (Korábek et al. 2022: fig. S59). That individual originated from the eastern margin of the presumed distribution range of *H.schlaeflii*. Although phylogenetically close to *H.pelagonesica*, the shell of the individual in question was different in shape and colouration from that species. This suggests that there is an as yet unknown lineage distinct from both *H.schlaeflii* and *H.pelagonesica*. But with only a single preserved individual and a handful of bleached shells, a proper assessment was not possible (this individual was referred to as *H.pelagonesica* by Korábek et al. 2022).

Due to this finding of a new lineage and the general scarcity of information on the distribution of *Helix* species in western Thessaly and the immediately adjacent regions, we decided to revise the eastern-most records of *H.schlaeflii* as reported by Neubert (2014: fig. 226). Three such sites were located in Thessaly (central Greece): Mouzaki, Morfovouni (the latter is the sampling site of the aforementioned sample) at the western margin of the Plain of Thessaly, and Loutropigi in the mountainous southwest of the region. Additionally, one site, Kastania near Servia, was in the south of West Macedonia (northern Greece). Beside this, we also revised records of *H.schlaeflii* from an isolated area at the southern tip of Sithonia, the middle “finger” of the Chalkidiki peninsula (northeastern Greece). These were presumably the eastern-most occurrences of the species.

As a result of the revisions, we describe a new, narrowly distributed subspecies of *H.pelagonesica* and provide an updated guide to the *Helix* species of the Greek mainland.

## ﻿Materials and methods

### ﻿Materials

This study is based on material collected in 2023 and stored in the Národní muzeum, Praha, Czechia and on data collected during our previous studies on *Helix* (e.g. Korábek et al. 2015), which included the examination of major museum collections (including the relevant type material, see Neubert 2014) and extensive fieldwork. The sources of re-used sequence data are listed in Suppl. material 1.

### ﻿Molecular phylogenetic analysis

For phylogenetic analyses we used sequences of fragments of three mitochondrial genes, the cytochrome c oxidase subunit I (*cox1*), 16S rRNA (*rrnL*) and 12S rRNA (*rrnS*). The fragments defined by the primers LCO1490+HCO2198 (*cox1*; as modified by Hausdorf et al. 2003), 16Scs1+16Scs2 (*rrnL*; Chiba 1999) and 12SGast_fwd2+12SGast_rev3 (*rrnS*; Cadahía et al. 2014) were sequenced in both directions. We sequenced samples from the alleged eastern-most localities of *Helixschlaeflii* and representative individuals of *H.schlaeflii* and two other Greek *Helix* species. In total, 12 samples were newly sequenced for this study.

Total genomic DNA was extracted from tissue samples following a slightly modified version of the protocol of Sokolov (2000) as detailed by Scheel and Hausdorf (2012). Parts of the mitochondrial cytochrome c oxidase subunit 1 gene (*cox1*), the 16S and the 12S rDNA were amplified by polymerase chain reaction (PCR) using the primer pairs LCO1490 plus HCO2198 (Folmer et al. 1994) for *cox1*, 12Sam plus 12Sbm (Hausdorf et al. 2003) and 16Scs1 plus 16Scs2 (Chiba 1999) for 12S and 16S rDNA, respectively. Amplifications were performed in 25 μl volumes containing 2.5 μl 10× DreamTaq Green Buffer (Thermo Fisher Scientific, Waltham, MA, USA), 1 μl dNTP mix (5 mM each, biolabproducts, Bebensee, Germany), 1 μl of each primer (10 μM), 0.2 μl DreamTaq DNA polymerase (5 U/μl; Thermo Fisher Scientific), 0.5 μl template DNA and 19.8 μl ultrapure H_2_O. The reaction conditions were 95 °C for 2 min, 35 PCR cycles 95 °C for 30 s, 48 °C (*cox1*, 16S rDNA) or 58 °C (12S rDNA) for 30 s, 72 °C for 1 min, followed by a final extension step at 72 °C for 7 min. Both strands of the amplified products were sequenced at Macrogen Europe (Amsterdam, The Netherlands).

In order to illustrate the phylogenetic placement of the analysed individuals, we combined the data generated here with available *Helix* sequences (Korábek et al. 2022) from mainland Greece and the neighbouring countries (Bulgaria, North Macedonia, and Albania; only species occurring also in Greece were included from those three countries). We used the three species of *Maltzanella* Hesse, 1917 (*M.dickhauti* (Kobelt, 1903), *M.maltzani* (Kobelt, 1883), *M.escherichi* (Boettger, 1898)) as an outgroup. The samples used are listed in Suppl. material 1 with the respective locality and voucher data and GenBank accession numbers.

Sequences were aligned with MAFFT 7.520 (Katoh and Standley 2013; --genafpair option). We performed partitioned Maximum likelihood analysis with IQ-TREE 2.3.6 (Minh et al. 2020). We partitioned the alignment into two rRNA genes and three codon positions of the *cox1* and selected substitution models with ModelFinder (part of IQ-TREE; Kalyaanamoorthy et al. 2017). We ran the tree search ten times and assessed the support for the tree with the best likelihood by 500 bootstraps.

### ﻿Morphological examination

Observations on anatomy of genital system of representatives of the *H.pelagonesica* clade were done on five individuals from Morfovouni, two individuals from the tip of Sithonia, and three individuals of typical *H.pelagonesica* from Nikiti (at the base of the Sithonia, Chalkidiki Peninsula). Neubert (2014: fig. 179) examined the genital system of a typical *H.pelagonesica* from the tip of Sithonia (Porto Koufo, ca 1200 m from where we sampled, mtDNA sequence included here). Measurements were performed on ethanol-preserved material in a Petri dish placed on a millimetre paper after gently stretching the measured parts of the genitalia.

**Figure 1.**
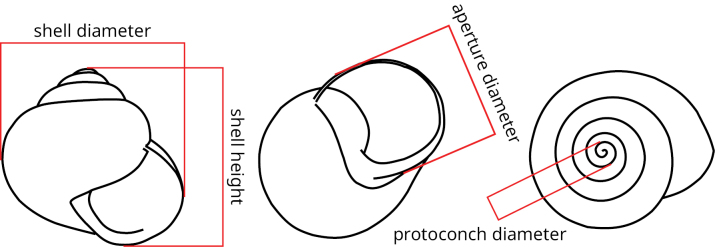
Scheme of the shell measurements taken on the shells of snails from the *Helixpelagonesica* clade.

We performed shell measurements to compare the three main lineages within the *H.pelagonesica* clade (typical *H.pelagonesica*, the one from Morfovouni and Mouzaki, and the lineage from the tip of Sithonia). With a calliper, we measured the shell diameter and height, the diameter of aperture (from the columellar margin to the insertion) and the diameter of protoconch at 1 whorl as counted by Kerney and Cameron (1979). In total, we measured 14 shells of the typical *H.pelagonesica* from two localities (Nikiti, Pigi), six shells from the tip of Sithonia, and 31 shells from Mouzaki and Morfovouni. The diameter of protoconch was measured to the nearest tenth of millimetre (but the repeatability was low, because finding a line perpendicular to the beginning of the suture is difficult), the other three measurements were rounded to the nearest millimetre. See Fig. 1 for a scheme of the measurements.

We performed a principal component analysis (PCA) on the data. We used the shell diameter to normalise the shell height and aperture diameter to size, which would otherwise dominate the first axis. The PCA was done in R 4.4 (stats:prcomp(x, center = TRUE, scale. = TRUE)) and visualised with the package ‘ggbiplot’ (Vu et al. 2024).

### ﻿Collection abbreviations

**ZMB**Museum für Naturkunde Berlin, Germany;

**ZMH**Zoological Museum Hamburg, Germany;

**NMBE**Naturhistorisches Museum der Burgergemeinde Bern, Switzerland;

**NMP** Národní muzeum, Praha, Czechia.

## ﻿Results

### ﻿New records and phylogenetic analysis

We could not confirm the presence of *H.schlaeflii* at any of the sites of its presumed eastern-most occurrences in Thessaly and Western Macedonia we visited (Neubert 2014; see Introduction). In the vicinity of Servia (including a site near Kastania), we only found *Helixphilibinensis* Rossmässler, 1839. However, the shells that Neubert (2014) referred to (NMBE 524741/2) and that should originate from Kastania are indeed *H.schlaeflii*. In Loutropigi, the only species we found was *Helixborealis* Mousson, 1859. Neubert’s material from this locality was not found. The sites in Morfovouni and Mouzaki are inhabited by the same form, which is the one previously sequenced and found to be related to *H.pelagonesica*. It is described here as a new subspecies, *H.pelagonesicathembones*. The snails from the tip of Sithonia were quite similar to the contrastingly coloured variant of *H.schlaeflii* from Corfu. However, their colouration was even more similar to (in fact indistinguishable from) *H.pelagonesica*, which also occurs on Sithonia and reaches near the tip (to Porto Koufo, which is only about a kilometre away; Neubert 2014; Korábek et al. 2015). The individual depicted in Neubert (2014: fig. 224) has unusually contrasting bands, the other material he cited is more similar to our samples. The main shell difference between the snails from the tip of Sithonia and *H.pelagonesica* is shape (higher whorls and thus also aperture, columella pointing more downwards), but this difference is not equally strong in all individuals.

The specimens from Krionéri in Aetolia-Acarnania (NMBE 524210), supposedly the southern-most locality of *H.schlaeflii*, have been found to be weathered *H.borealis*, based on the shape of the columellar triangle and the size of the protoconch. We did not visit that locality. Besides revisiting the identity of the species occurring at the localities mentioned above, we found *Helixstraminea* Briganti, 1825 near Kalambaka, a species occurring in Albania but as yet not reported from Greece. We also extended the area from where *Helixthessalica* Boettger, 1886 is known in Greece westward by confirming its presence near Livadia in the massif of Mount Paiko/Pajak in the north of the country.

**Figure 2.**
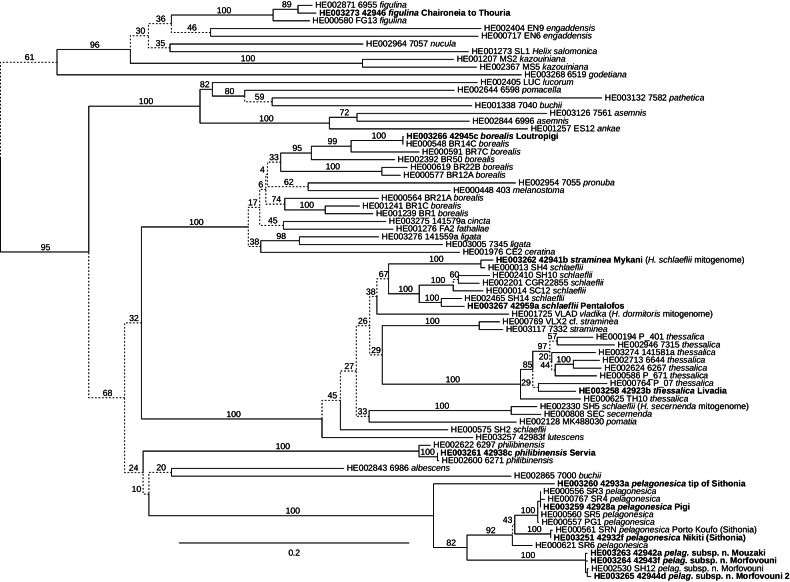
Phylogenetic relationships of the newly analysed *Helix* samples from Greece (in bold). Maximum likelihood tree based on concatenated mitochondrial nucleotide sequences of partial *cox1*, *rrnL*, and *rrnS* genes. Support values are bootstrap percentages from 500 pseudoreplicates. Branches with bootstrap support <75% are dashed. The tree is rooted with *Maltzanella* Hesse, 1917; the outgroup is not shown.

Phylogenetic analysis of mitochondrial sequences (Fig. 2) placed the samples of *H.philibinensis* from Servia and *H.borealis* from Loutropigi to already known clades in agreement with their geographic origin. The *H.thessalica* sample from Mount Paiko turned out to be a new major intraspecific lineage, additional to those reported by Korábek et al. (2022) and included in Fig. 2. In the case of the *H.straminea* sample, the recovered sequence did not match the species identity and belonged to a clade characteristic for Albanian *H.schlaeflii*. However, this mitochondrial lineage was already recorded from *H.straminea* before in Albania (Orenjë near Librazhd; Korábek et al. 2022: fig. S38) and the identification of the species based on shell characters is unambiguous.

The samples from Mouzaki and Morfovouni yielded sequences nearly identical to the previously analysed sample from the latter locality. They form a branch sister to typical *H.pelagonesica*. This clade is then sister to a new mitochondrial lineage recovered from the sample from the tip of Sithonia.

### ﻿Shell shape differentiation within the clade of *H.pelagonesica*

**Figure 3.**
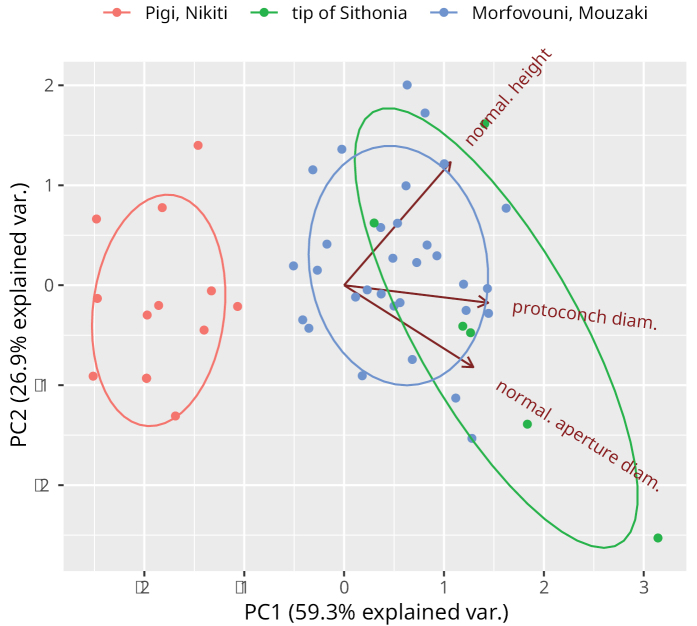
Principal component analysis of the variation in shell height, aperture diameter and protoconch size among typical *Helixpelagonesica* and the two related lineages (all normalised with respect to the diameter, thus removing the effect of shell size).

**Figure 4.**
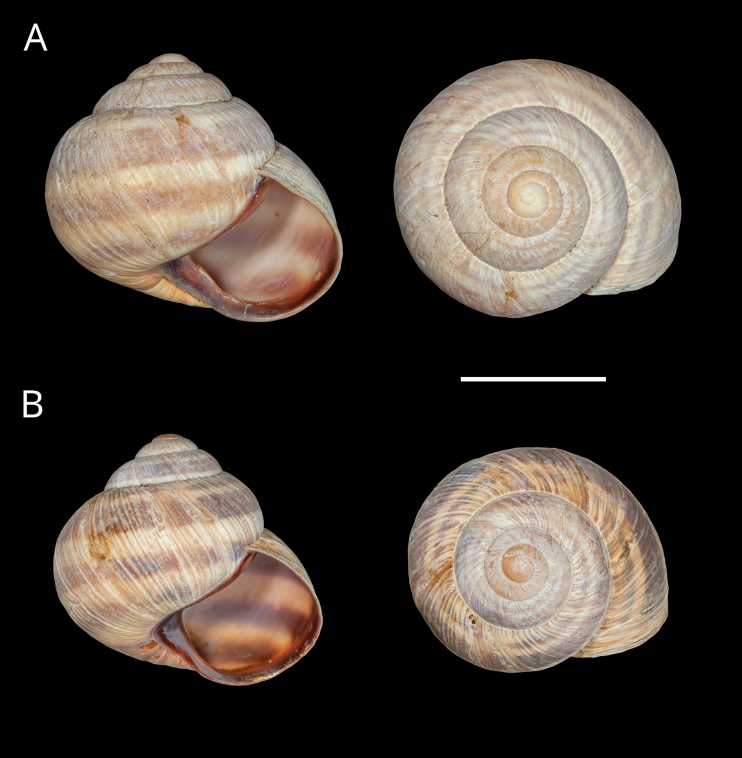
Shells of typical *Helix (Helix) p. pelagonesica.* A. Greece, Central Macedonia, Pigi; 41.0148°N, 22.4865°E; NMP P6M 42928; B. Greece, Central Macedonia, Nikiti; 40.2058°N, 23.6975°E; NMP P6M 42932). Photo R. Coufal. Scale bar: 2 cm.

Of the three lineages, typical *H.pelagonesica* is characterised by shells with a more triangular outline from the frontal view (Figs 3, 4), in contrast to a more globular shape in the other two (in particular that from Morfovouni and Mouzaki). This is because the columellar margin of the aperture is more oblique than in the other two (especially than the form from the tip of Sithonia) and the body whorl and aperture are lower relative to the shell height. The diameter of the protoconch and the relative height of the shell and relative size of the aperture tend to be smaller in the examined typical *H.pelagonesica* (Fig. 3).

### ﻿Systematics and a guide to mainland Greek *Helix*

#### ﻿Family Helicidae

Taxon classificationAnimaliaStylommatophoraHelicidae

Rafinesque, 1815

16294ED5-87C7-5DD4-B93A-9695E52F01FC

##### References.

Schileyko 1978; Nordsieck 1987; Razkin et al. 2015.

##### Type genus.

*Helix* Linnaeus, 1758

#### ﻿Subfamily Helicinae

Taxon classificationAnimaliaStylommatophoraHelicidae

Rafinesque, 1815

E8F21491-9FE6-5BA7-9929-E5A2E592E57D

##### References.

Hesse 1918; Schileyko 1978; Razkin et al. 2015.

#### ﻿Tribe Helicini

Taxon classificationAnimaliaStylommatophoraHelicidae

Rafinesque, 1815

2F68F9EC-1CB5-5F17-843C-7750C5221C0B

##### References.

Razkin et al. 2015; Neiber et al. 2022.

#### ﻿Genus Helix

Taxon classificationAnimaliaStylommatophoraHelicidae

Linnaeus, 1758

3663380B-9586-5A60-9440-9C18574CBF31

##### References.

Hesse 1908; Giusti et al. 1995; Neubert and Bank 2006; Korábek and Hausdorf 2023).

##### Type species.

*Helixpomatia* Linnaeus, 1758, by subsequent designation (de Montfort 1810: 231).

#### ﻿Subgenus Helix

Taxon classificationAnimaliaStylommatophoraHelicidae

Linnaeus, 1758

285A409F-94F1-5D17-A9B9-89E5D43AC2AD

##### References.

Hesse 1908; Neubert 2014; Korábek and Hausdorf 2023.

#### ﻿ Helix (Helix) pelagonesicapelagonesica

Taxon classificationAnimaliaStylommatophoraHelicidae

(Rolle, 1898)

393122E9-46E4-5C2F-8F51-AFF7F91040D8

**Figure 5.**
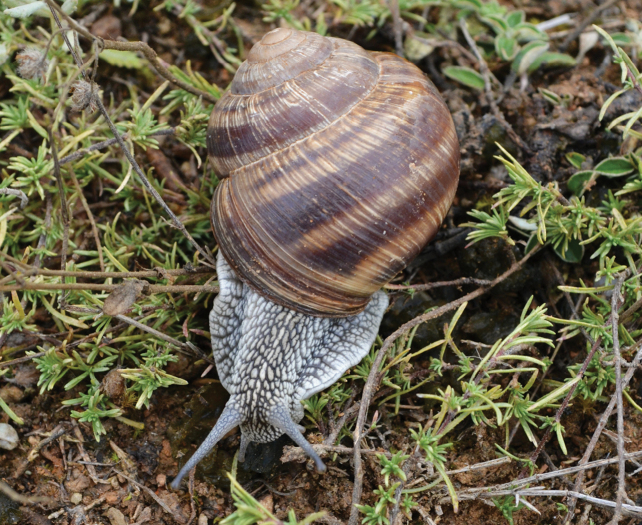
Helix (Helix) p. pelagonesica (Greece, Central Macedonia, Petra Olympou; 40.1803°N, 22.3286°E; SMF 342487). Photo O. Korábek.

**Figure 6.**
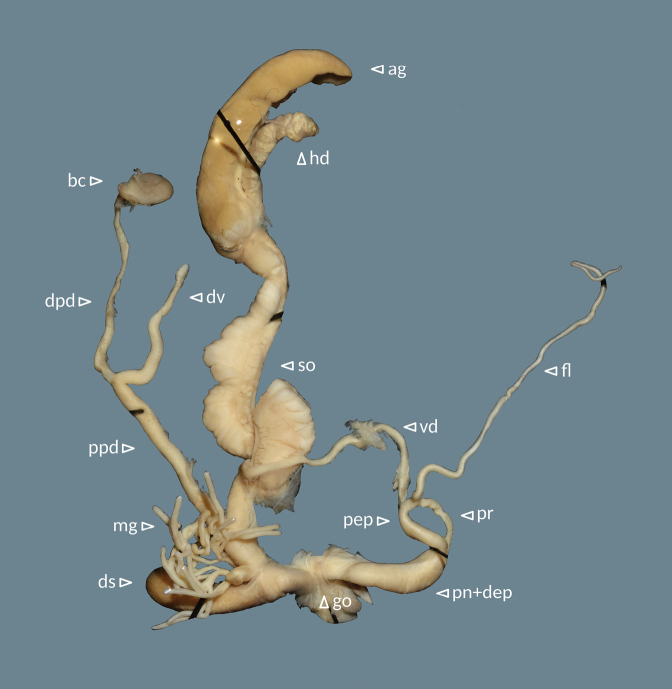
Genital system of Helix (Helix) p. pelagonesica (Greece, Central Macedonia, Nikiti; 40.2058°N, 23.6975°E; NMP P6M 42932). Abbreviations: ag = albumen gland; bc = bursa copulatrix; dpd = distal pedunculus; ds = dart sac; dv = diverticulum; fl = flagellum; go = genital opening (not visible); hd = hermaphroditic duct; mg = mucous glands; pn+dep = penis + distal epiphallus; pep = proximal epiphallus; ppd = proximal pedunculus; pr = penial retractor muscle; so = spermoviduct; vd = vas deferens.

**Figure 7.**
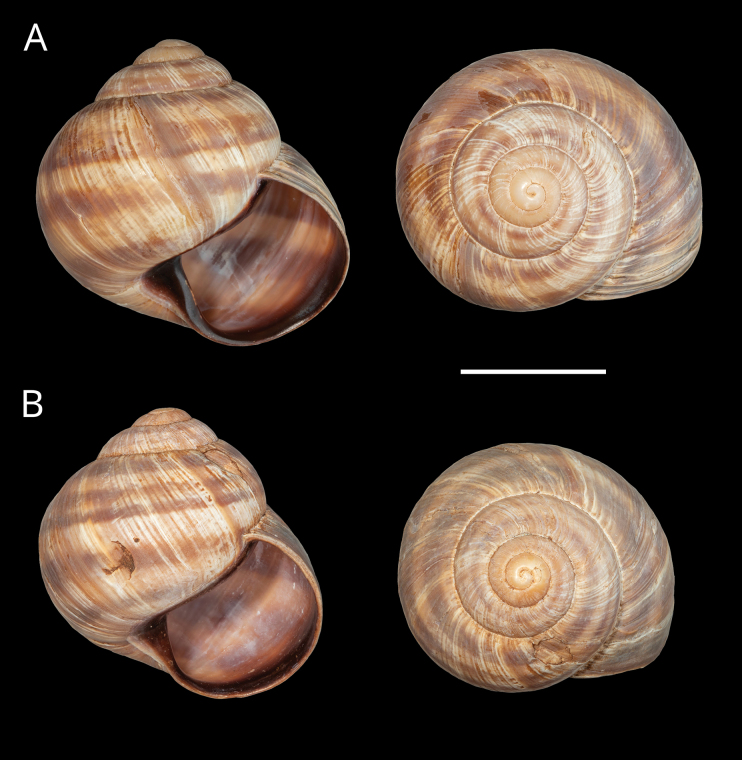
Shells of Helix (Helix) p. pelagonesica from the tip of Sithonia (Greece, Central Macedonia, Toroni; 39.9476°N, 23.9321°E; NMP P6M 42933). A few weathered shells were collected at the site as well, some showing even greater height-to-diameter ratio. Photo R. Coufal. Scale bar: 2 cm.

**Figure 8.**
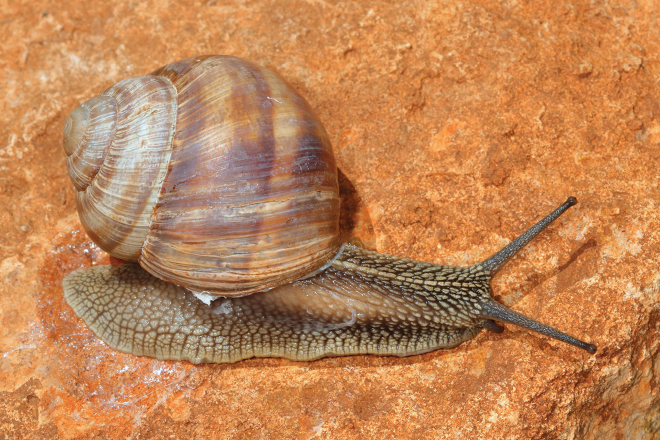
Helix (Helix) p. pelagonesica from the tip of Sithonia (Greece, Central Macedonia, Toroni; 39.9476°N, 23.9321°E; NMP P6M 42933). Photo R. Coufal.

**Figure 9.**
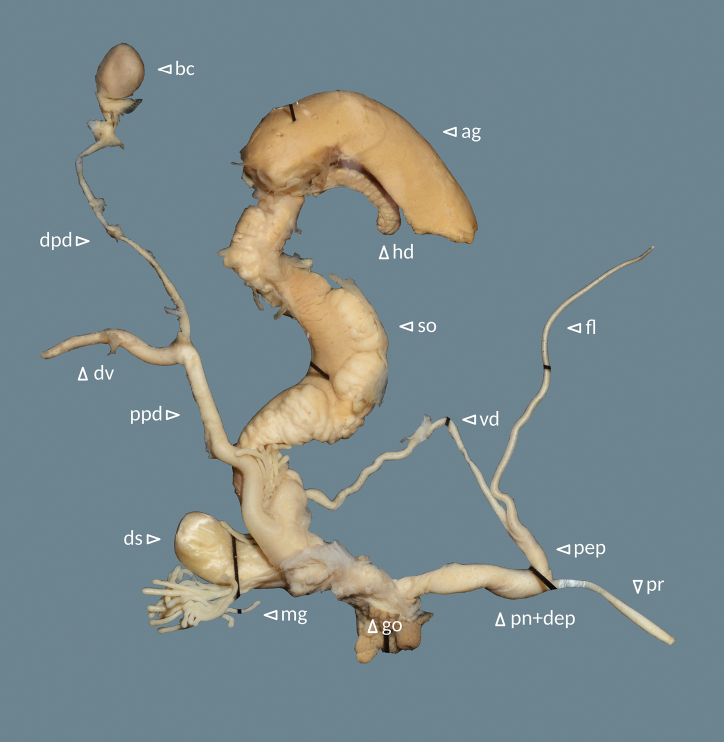
Genital system of Helix (Helix) p. pelagonesica from the tip of Sithonia (Greece, Central Macedonia, Toroni; 39.9476°N, 23.9321°E; NMP P6M 42933). Abbreviations: ag = albumen gland; bc = bursa copulatrix; dpd = distal pedunculus; ds = dart sac; dv = diverticulum; fl = flagellum; go = genital opening (not visible); hd = hermaphroditic duct; mg = mucous glands; pn+dep = penis + distal epiphallus; pep = proximal epiphallus; ppd = proximal pedunculus; pr = penial retractor muscle; so = spermoviduct; vd = vas deferens.

Figs 4 , 5 , 6 , 7 , 8 , 9

##### References.

Neubert 2014; Korábek et al. 2022.

##### Description.

Shell (Fig. 4) middle-sized, conical with relatively flat base; individual whorls low and tightly coiled; deep suture giving the shell a stepped look; umbilicus fully covered or there is very narrow slit between the shell bottom and the reflected columellar margin of the aperture; protoconch large, making the apex blunt; aperture small and low, with oblique columella; aperture margins and the parietal area dark brown with purple hue; shell surface usually with fine ribs, pale brown, but often corroded even in live individuals; four bands typically present (because 2+3 fuse), brown with reddish or purple tones; bands interrupted by fine growth lines and lightly coloured ribs; aperture margins straight, columellar margin thickened, margins and the parietal area dark purple-brown. Animal grey (Fig. 5).

##### Distribution and habitat.

**Figure 10.**
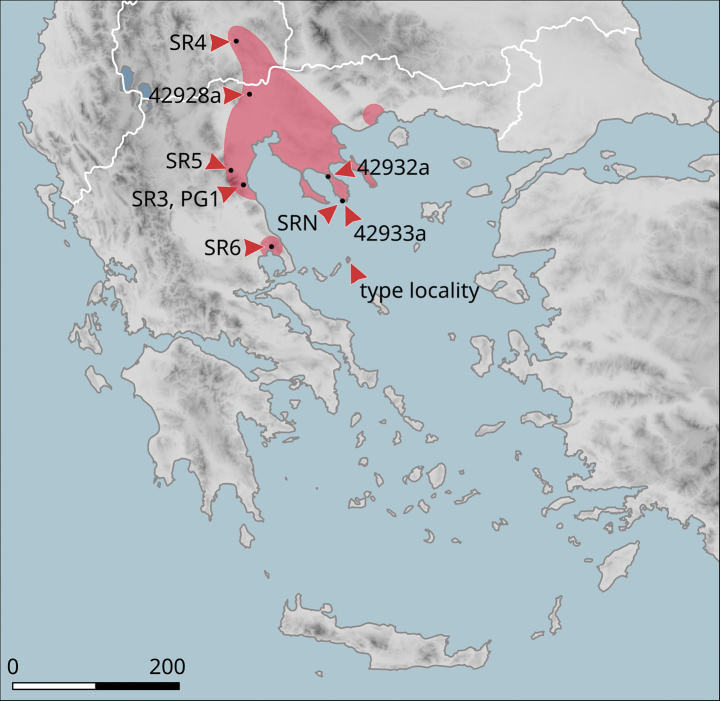
Approximate distribution of Helix (Helix) p. pelagonesica. Black dots denote populations sampled for molecular analyses. Isolate codes as in the phylogeny in Fig. 2 are shown for reference and the position of the island of Kyra Panagia (= Pelagonisi), the type locality of *H.pelagonesica*, is indicated.

It has a small range (Fig. 10), within which it occurs sporadically, even though it can be locally abundant. In the north, it was found in the hills on both sides of the valley of Vardar/Axios (Kilkis Regional Unit), e.g. west of Polykastro or near the Dojran Lake. The northernmost record is from northeast of Negotino in North Macedonia. To the south, *H.p.pelagonesica* is distributed over Chalkidiki, additional populations are known from foothills of Mount Olympus and from Pelion near Volos (in Makrinitsa). The type locality is the island of Kyra Panagia (Pelagonisi) in the Northern Sporades. The characteristic habitat is a loose shrub with *Quercus* (*Q.coccifera* or similar species) and *Paliurusspina-christi*. It seems to rest relatively frequently on branches of shrubs.

##### Remarks.

Neubert (2014: fig. 224) figured a shell of a snail from the tip of the Sithonia peninsula, Chalkidiki (NMBE 524743), which was quite similar to the brightly coloured form of *H.schlaeflii* from Corfu. He discussed this population with the first author in 2011 and back then both eventually identified it as *H.schlaeflii*, speculating it was possibly an anthropogenic occurrence. Following the examination of new material and phylogenetic analysis (Fig. 1), we currently consider the form living at the tip of Sithonia to be a variety of *H.p.pelagonesica*. Its globular shell shape is a clear difference to typical *H.p.pelagonesica*, but this is a question of the height of individual whorls driven probably by the angle of the columella, so a change in a single parameter of the shell geometry is responsible for the difference. In some shells, the columella is not vertical and the difference in shell shape is much weaker (whorls are lower, suture deeper). The colouration of the shell and animal is identical to typical *H.p.pelagonesica*, whose sample was previously sequenced from a locality on the northern foot of a limestone hill near Porto Koufo (just over a kilometre from where we collected). The strongest argument for a separate taxon for the snails from the tip of Sithonia is the mitochondrial lineage found there, which does not fall within or sister to the clade comprising typical *H.p.pelagonesica* samples. However, the conchological similarities as well as geographic proximity suggest that there is a gene flow; the divergent mtDNA might be a relic of a period of separation that was then followed by merging back with *H.p.pelagonesica*. We found this form (Figs 7, 8) on the limestone hills on both sides of the Maratia Beach at the southernmost tip of the peninsula. This habitat contrasts with geological conditions on the rest of Sithonia, which is covered by substrates generally unfavourable for snails, in large part by granite and other plutonic rocks (Christofides et al. 2007). However, a strict dependence on limestone is unlikely, because Neubert (2014) also found a locality of the same form north of the limestone occurrences (17 road km south of Sarti, ~39.993°N, 23.959°E; NMBE 524745). Typical *H.p.pelagonesica* tolerates non-limestone substrates.

#### ﻿ Helix (Helix) pelagonesicathembones

Taxon classificationAnimaliaStylommatophoraHelicidae

Korábek, Juřičková & Hausdorf subsp. nov.

4B4C8C92-A925-5699-BF32-CDACC2C95420

https://zoobank.org/0CEEF1A3-6DF5-4961-91C2-34A49415CC19

Figs 11 , 12 , 13

##### Type material.

**Figure 11.**
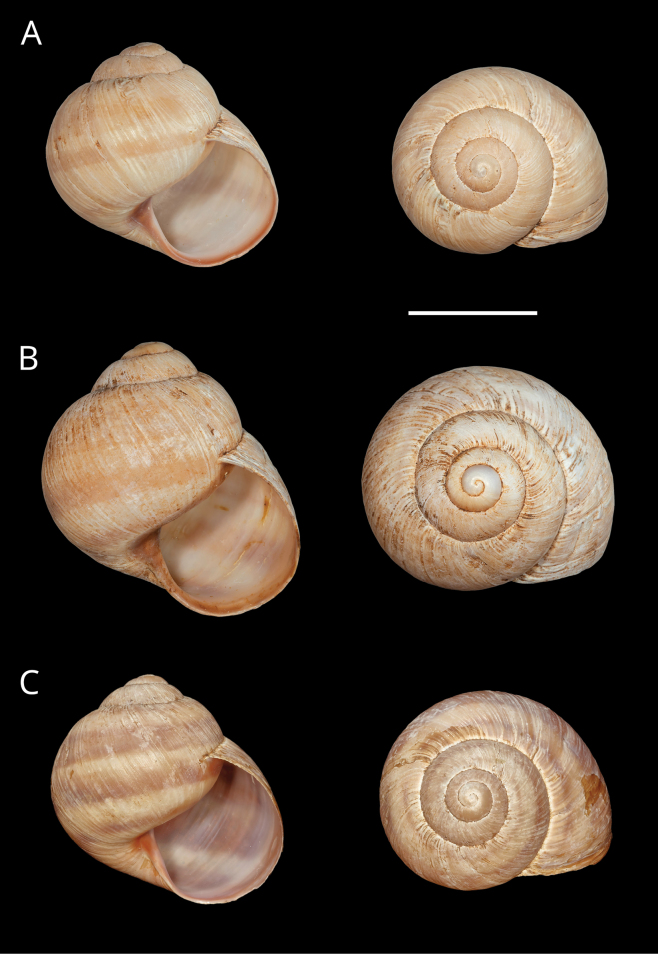
Shells of *Helix (Helix) pelagonesica thembones*. A. Holotype; Greece, Thessaly, Morfovouni; 39.3574°N, 21.7468°E; NMP P6M 44038; B. Greece, Thessaly, Mouzaki; 39.4135°N, 21.6642°E; NMP P6M 42942; C. Greece, Thessaly, between Morfovouni and Ellinopyrgos; 39.3747°N, 21.7318°E; NMP P6M 42944. Photo R. Coufal. Scale bar: 2 cm.

***Holotype***: (Fig. 11A) Greece • diameter 36 mm, height 37 mm; Thessaly, Morfovouni, hill on the NW outskirts of the village; 39.3574°N, 21.7468°E; 15 Apr. 2023; O. Korábek et al. leg.; NMP P6M 44038. The type locality is a northern side of the top of a small hill at the northwestern margin of Morfovouni (Μορφοβούνι; formerly Βουνέσι), Karditsa regional unit, Thessaly (Θεσσαλία). ***Paratypes***: Greece • 15 shells, 9 bodies in ethanol; Thessaly, Morfovouni, hill on the NW outskirts of the village; 39.3574°N, 21.7468°E; 15 Apr. 2023; O. Korábek et al. leg.; NMP P6M 42943 • 2 shells; Morfovouni, hill on the NW outskirts of the village; 39.3574°N, 21.7468°E; 15 Apr. 2023; O. Korábek et al. leg.; ZMH 141525 • 12 shells; south of Mouzaki, by a road to Porti; 39.4135°N, 21.6642°E; 15 Apr. 2023; O. Korábek et al. leg.; NMP P6M 42942 • 6 shells; between Morfovouni and Ellinopyrgos, slope above a forest road; 39.3747°N, 21.7318°E; 16 Apr. 2023; O. Korábek et al. leg.; NMP P6M 42944 • 1 shell; rocks northwest of Morfovouni; 18 Jun.1985; B. Hausdorf leg.; ZMH 137861 • 1 shell; Morfovouni, crystallic rocks; 18 May 1995; P. Subai leg.; NMBE 524759 • 3 shells; Morfovouni, northern fringes, limestone rocks; 12 May 1997; P. Subai & M. Szekeres leg.; NMBE 524756 • 1 complete individual in alcohol; Morfovouni, northern fringes, east-oriented limestone rocks; 39.3567°N, 21.7464°E; 23 Apr. 2003; P. Subai leg.; NMBE 524760 • 1 shell; Mouzaki, rocks 1.5 km towards Kryonéri; 19 Jun. 1985; B. Hausdorf leg.; ZMH 137863 • 1 shell; 800 south of Mouzaki, junction to Porti; 39.4153°N 21.6659°E; 10 Apr. 1988; P. Subai leg.; NMBE 524757 • 1 shell; 1.6 km south of Mouzaki, junction to Porti; 39.4113°N 21.6630°E; 23 Apr. 2003; P. Subai leg.; NMBE 524746 • 1 shell; Elati, 7.3 km in direction to Pertouli and 2.9 km east on field road, coniferous forest, on limestone rocks; 39.5710°N, 21.5179°E; 23 Jul. 1990; P. Subai leg.; NMBE 524754 • 1 shell; Elati, 7.3 km in direction to Pertouli and 2.9 km east on field road, coniferous forest, on limestone rocks; 39.5710°N, 21.5179°E; 17 May 1991; P. Subai leg.; NMBE 524755 • 1 shell; Elati, 7.3 km in direction to Pertouli and 2.9 km east on field road, coniferous forest, on limestone rocks; 39.5710°N, 21.5179°E; 16 May 1995; P. Subai leg.; NMBE 524754.

##### Diagnosis.

*Helixpelagonesicathembones* differs from *H.p.pelagonesica* in the pale brownish shell with inconspicuous bands and paler apertural margins. *Helixpelagonesicathembones* has globular shells, whereas most populations of *H.p.pelagonesica* have more conical shells with a relatively smaller aperture.

**Figure 12.**
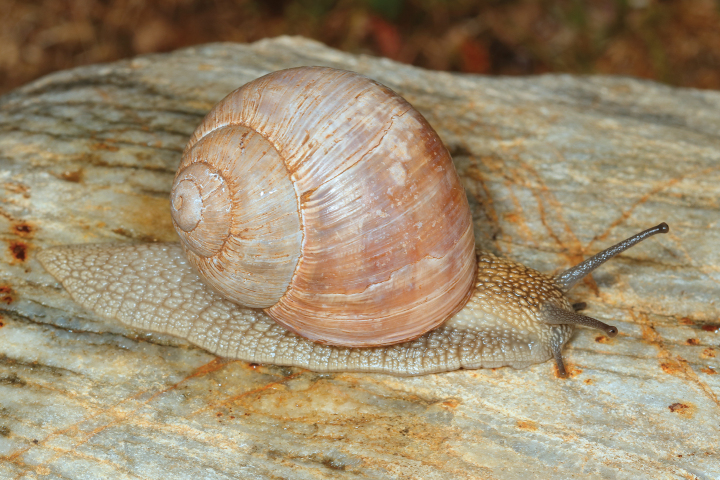
Helix (Helix) pelagonesica thembones (Greece, Thessaly, Morfovouni; 39.3574°N, 21.7468°E). Photo R. Coufal.

##### Description.

Middle-sized globular shell (diameter 32–42 mm, height 33–43 mm) resembling in shape *Helixpomatia*; no umbilicus; protoconch large (~5–6 mm in diameter at 1 whorl); aperture semicircular; aperture margins and especially columella may be darker than the rest of the shell, with columella often meat-coloured to brown; shell surface relatively smooth, with only fine irregular riblets, pale brownish, sometimes with pinkish hue when alive; bands are inconspicuous, not much darker than the background, but present (2+3 and 4, sometimes weakly also 5, positioned close to the shell axis); apertural margins straight, slightly reflected only towards columella; animal pale brown to greyish brown, mantle margins pale.

**Figure 13.**
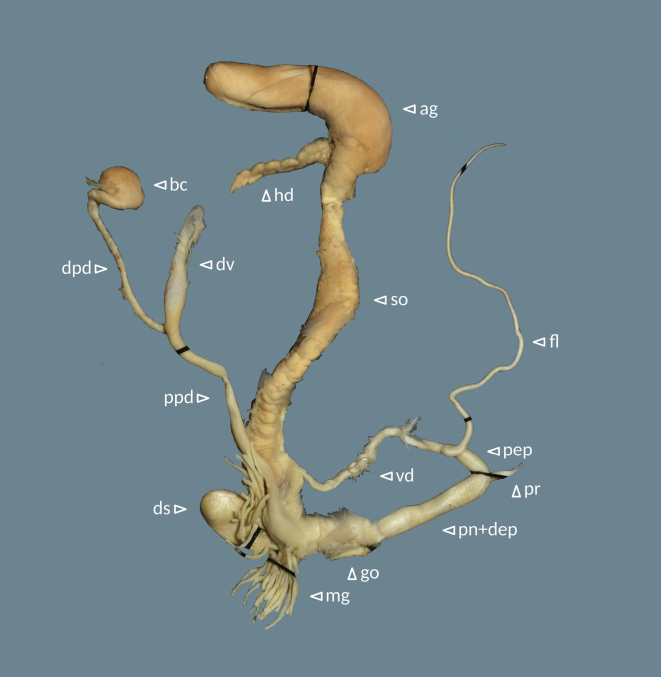
Genital system of Helix (Helix) pelagonesica thembones (paratype; Greece, Thessaly, Morfovouni; 39.3574°N, 21.7468°E; NMP P6M 42943). Abbreviations: ag = albumen gland; bc = bursa copulatrix; dpd = distal pedunculus; ds = dart sac; dv = diverticulum; fl = flagellum; go = genital opening (not visible); hd = hermaphroditic duct; mg = mucous glands; pn+dep = penis + distal epiphallus; pep = proximal epiphallus; ppd = proximal pedunculus; pr = penial retractor muscle; so = spermoviduct; vd = vas deferens.

Genital system (Fig. 13) with proximal epiphallus (sensu Korábek and Hausdorf 2023) much shorter than distal epiphallus and penis combined (4–5 vs 12–15 mm); flagellum well-developed (38–49 mm), mucous gland with many branches and of similar length as or longer than the dart sac; distal pedunculus of bursa copulatrix considerably thicker than the proximal part and leading to a well-developed diverticulum; diverticulum shorter than the proximal pedunculus (12–17 vs 21–32 mm), thick and flattened, narrowing towards tip (heavily swollen in the individual with a spermatophore in the pedunculus); distal genitalia (penis, epiphallus, vagina, the distal-most pedunculus) white.

##### Etymology.

Named after the opening track of the Alice in Chains’ 1992 album “Dirt” as a little reminder to all in power that they are also “gonna end up a big ol’ pile of them bones”. Noun in apposition.

##### Distribution.

**Figure 14.**
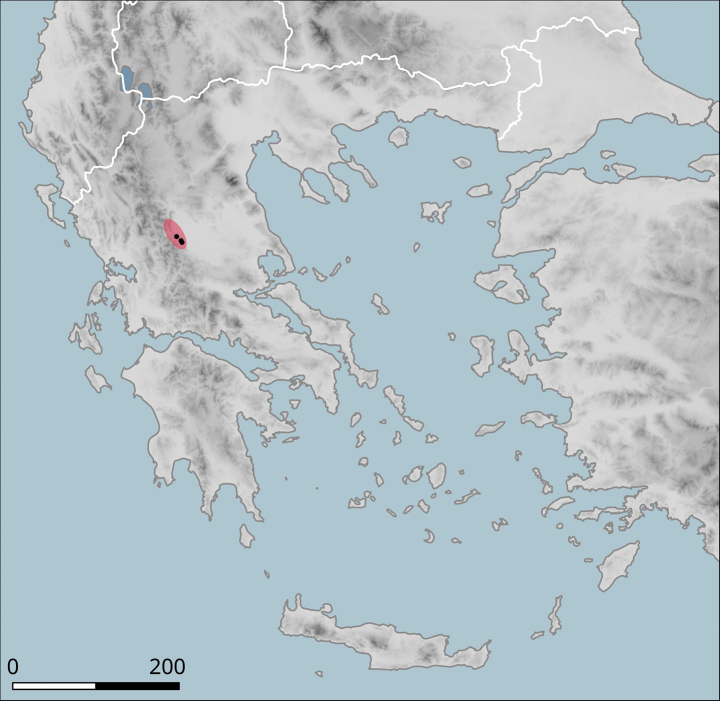
Approximate distribution of Helix (Helix) pelagonesica thembones. Black dots denote populations sampled for molecular analyses.

The species was found on the band of hills that directly adjoin the Plain of Thessaly from the west (Fig. 14). The southern-most known locality is Morfovouni, the northernmost is ca 7 km north of Elati, so the known range extent is only slightly more than 30 km.

##### Ecology.

**Figure 15.**
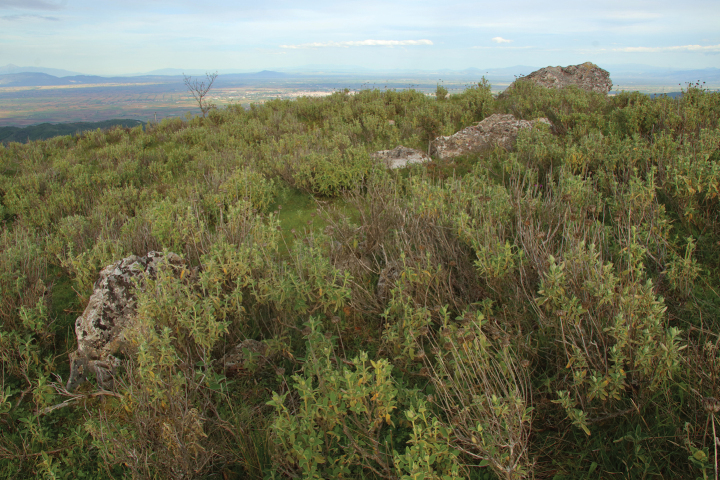
Limestone hill in Morfovouni, the type locality of Helix (Helix) pelagonesica thembones (Greece, Thessaly; 39.3574˚N 21.7468˚E; 15.4.2023). Photo R. Coufal.

At the type locality, the greatest concentration of individuals was in an area overgrown by *Phlomisfruticosa* L. (Fig. 15). The same plant dominated vegetation in a small clearing in an oak forest northwest of Morfovouni, where we also found the species. The biology of the subspecies has not been studied. A spermatophore found in the bursa pedunculus of one of the dissected individuals indicates that mating takes place (at least partially) in April.

##### Remarks.

The majority of the *Helixpelagonesica* populations differ in a conical shell shape from the plesiomorphic more globular shell shape in *Helix*. This conical shell shape characterises not only the populations in the contiguous main range of the species in Greek Macedonia and North Macedonia, for which the name *Helixpelagonesicavardarica* Knipper, 1939 has been proposed, but also isolates of the species in Thessalia, for which the name *Helixvolensis* Kobelt, 1906 was proposed, and the population from the island Kyra Panagia (= Pelagonisi) in the Northern Sporades, for which the species name was originally proposed. The populations with conical shell shape are monophyletic in the mitochondrial tree (Fig. 2). They are hardly differentiated. Therefore, the names given to these populations were synonymised (Neubert 2014). In contrast, there are populations from the surroundings of Morfovouni in western Thessaly and from the tip of Sithonia, the middle “finger” of the Chalkidiki peninsula in Macedonia, which differ from other populations of *Helixpelagonesica* so strongly in a globular shell shape that Neubert (2014) misidentified them as *H.schlaeflii*. It turned out that these globular populations are not related to *H.schlaeflii* (Fig. 2) but are the next relatives of *H.pelagonesica*. They were obviously isolated from the main lineage of *H.p.pelagonesica* before its shell became conical. However, the globular populations had different fates. There is probably no gene flow between the populations in the isolate in western Thessaly and those in the main range of the species because of the large geographic distance. This is supported by the distinctive pale shell of these populations (compare Figs 4, 5 to Figs 11, 12). Therefore, we suggest separating the populations from western Thessaly as a distinct subspecies *Helixpelagonesicathembones* from the other populations of *Helixpelagonesica*.

In contrast, the population from the tip of Sithonia came probably in secondary contact with the conical populations when they colonised the rest of Sithonia. We suppose that the globular population from the tip of Sithonia is connected by gene flow with the neighbouring *H.pelagonesica* populations, which are only about a kilometre apart, and that the differentiated populations are in the process of merging. Therefore, we currently classify this population as *H.p.pelagonesica* despite the still recognisable difference in shell shape and the deep split between the mitochondrial lineages of these populations (Fig. 2). The relationships between the *H.pelagonesica* populations should be re-examined by multi-locus markers. If it would turn out that gene flow between the globular population from the tip of Sithonia and the neighbouring populations remained restricted despite their proximity and that their genetic differentiation based on the multi-locus data reflects still the deep split between their mitochondrial lineages, it should be re-considered to classify also the population from the tip of Sithonia as a distinct subspecies.

We did not find any noteworthy differences between the genital system of *H.pelagonesicathembones*, typical *H.pelagonesica* and the form from the tip of Sithonia. The latter might have a somewhat shorter flagellum (35–37 mm, *n* = 2) compared to *H.pelagonesicathembones* (38–49 mm, *n* = 5), but both overlap with the typical *H.pelagonesica* (35–45 mm, *n* = 3).

*Helixpelagonesicathembones* is usually smaller than *H.schlaeflii* and has a smoother shell surface without the irregular whitish patterns typical for the latter. It differs from individuals of *H.borealis* with reduced banding in much paler colouration of the aperture margins and a much larger protoconch. Furthermore, the mucous glands in *H.borealis* are shorter, reaching only to the half of the dart sac, and diverticulum of bursa copulatrix may be much shorter in some individuals. Compared to *H.philibinensis*, which is usually distinctly banded, *H.pelagonesicathembones* has a higher aperture.

#### ﻿ Helix (Helix) schlaeflii

Taxon classificationAnimaliaStylommatophoraHelicidae

Mousson, 1859

263DCD36-E7BA-5EBB-B576-733FE6049051

Figs 16 , 17

##### References.

Neubert 2014; Korábek et al. 2022.

##### Description.

Shell large, globular to conical; umbilicus sometimes slit-like but usually completely covered; protoconch large; shell surface with irregular ribs; basal colour whitish or very pale brown, more rarely the whole shell is brown; banding often reduced with upper bands 2 and 3 fused or partially fused and the lower two fuzzy and faint, but some populations have well developed, contrasting, reddish brown bands; bands with irregular whitish interruptions; aperture margins straight, only slightly reflected towards the columella; apertural lips and in particular the columellar triangle orange- or meat-brown to violet-brown, but the colouration is sometimes only faint and is missing or only weakly developed in the parietal area; mantle margins pale; animal very pale brown or grey to yellowish.

**Figure 16.**
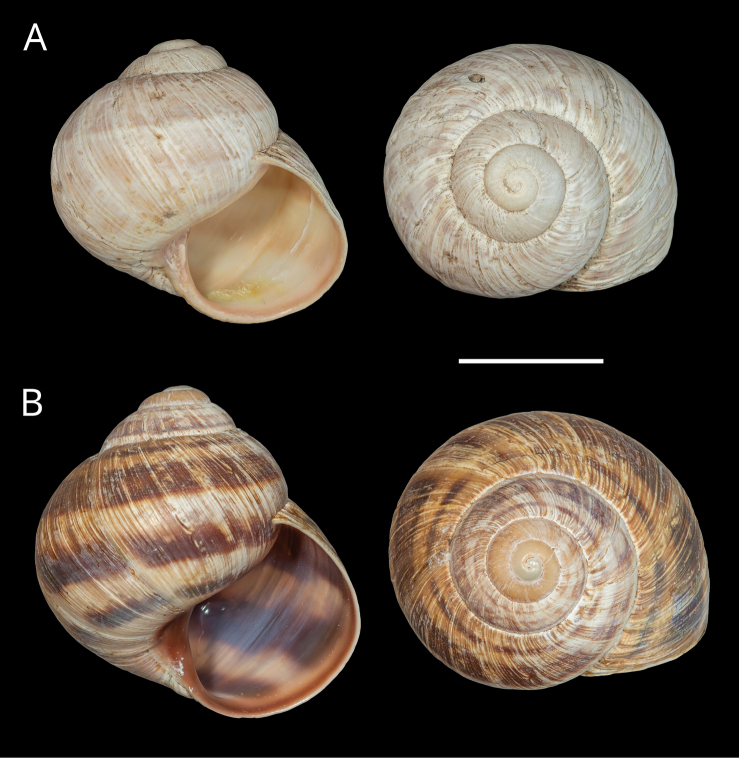
Shells of Helix (Helix) schlaeflii. A. Greece, Western Macedonia, pass between Eptachori and Pentalofos; 40.2048°N, 21.0951°E; NMP P6M 42959; B. Albania, Gjirokastër County, Jorgucat; 39.9391°N, 20.2609°E; NMP P6M 42964. Photo R. Coufal. Scale bar: 2 cm.

**Figure 17.**
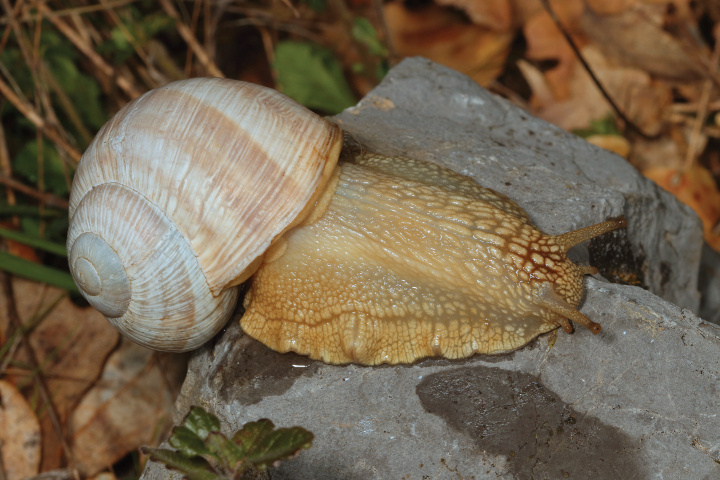
Helix (Helix) schlaeflii (Greece, Western Macedonia, between Koryfi and Chrysavgi; 40.1757°N, 21.2302°N). Photo R. Coufal.

##### Distribution and habitat.

**Figure 18.**
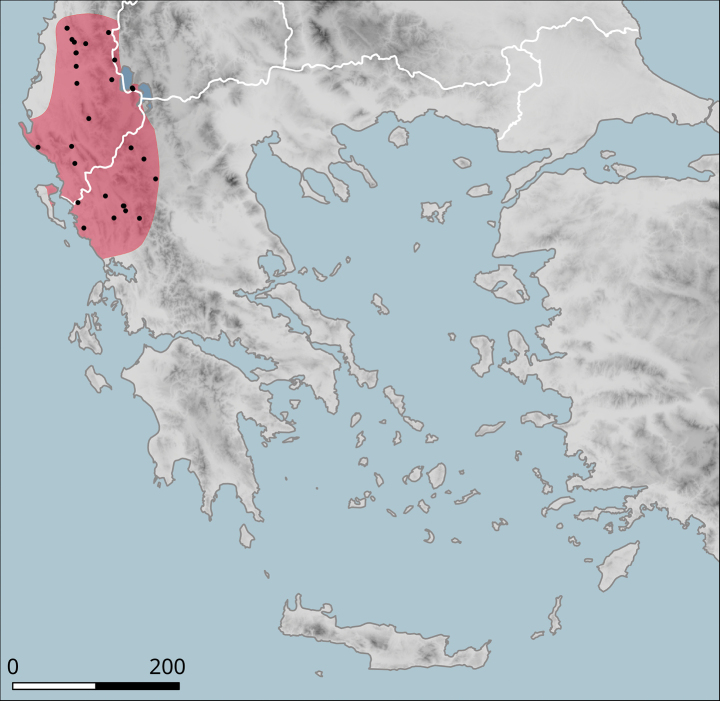
Approximate distribution of Helix (Helix) schlaeflii. Black dots denote populations sampled for molecular analyses.

Common in Epirus and adjacent Western Macedonia (eastern limits uncertain, but apparently west of Kastoria, Neapoli and Grevena); occurs also on Kerkyra (Corfu). The range of the species as currently accepted extends to central Albania and up to the Galičica Mountains between the lakes Ochrid and Prespa (Fig. 18). In Greece, it occurs in a range of habitats, from Mediterranean-type shrubby vegetation on limestones in low altitudes in the west to more temperate landscapes on sandstones in the east, but occurs also in pine forests as well as on the margins of beech forests in altitudes over 1000 m. Geophilous.

##### Remarks.

Differences to *H.thessalica* and *H.borealis* are described under the respective species. *Helixschlaeflii* differs from *H.straminea* in globular shape with an expanded last whorl and a larger aperture; the Greek populations often differ in a pale colouration, because *H.straminea* has typically more vivid colours. *Helixstraminea* also lacks the whitish pattern on the bands characteristic for *H.schlaeflii* and often has a darker, brown foot.

*Helixschlaeflii* is not monophyletic in the mitochondrial tree (Fig. 2). This is due to three issues. First, there are two mitochondrial clades in this species that are deeply divergent and whose mutual relationships remain unresolved. Second, the population from Krujë (central Albania, isolate SH5 in the tree) possesses a mitochondrial lineage introgressed from *Helixsecernenda* Rossmässler, 1847. Third, a sample of *H.straminea* yielded a mitochondrial lineage typical for *H.schlaeflii*, apparently also due to introgression. Mitochondrial introgressions are not rare in the western-Balkan radiation of *Helix* (Korábek et al. 2022) and an ancient introgression may in fact account also for the presence of two divergent lineages within *H.schlaeflii*. Introgressions are generally a major problem for species identification using mitochondrial data (Funk and Omland 2003).

#### ﻿ Helix (Helix) straminea

Taxon classificationAnimaliaStylommatophoraHelicidae

Briganti, 1825

5FD85ABD-F58E-5022-81D6-37369BCE86E1

Figs 19 , 20

##### References.

Korábek et al. 2014, 2022; Petraccioli et al. 2021.

##### Description.

**Figure 19.**
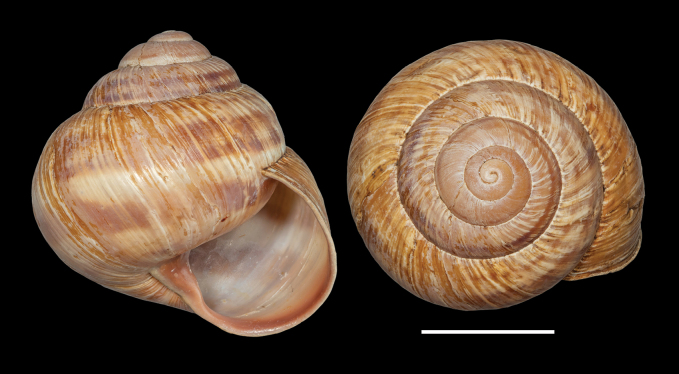
Shell of Helix (Helix) straminea (Greece, Thessaly, Mykani northeast of Kalambaka; 39.7964°N, 21.5315°E; NMP P6M 42941). Photo R. Coufal. Scale bar: 2 cm.

**Figure 20.**
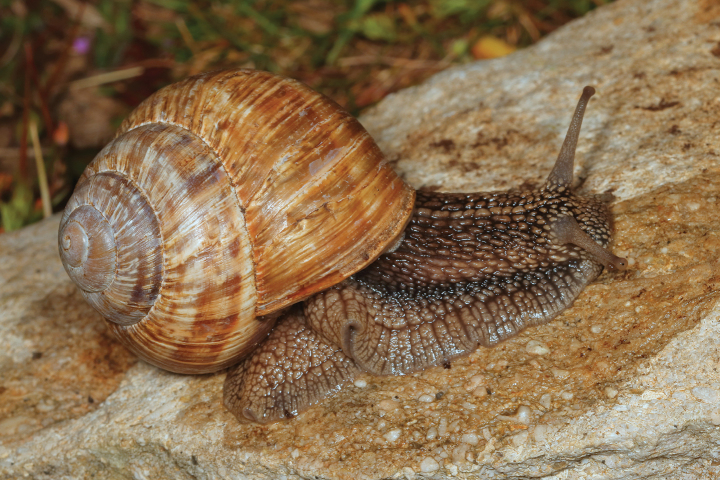
Helix (Helix) straminea (Greece, Thessaly, Mykani northeast of Kalambaka; 39.7964°N, 21.5315°E). Photo R. Coufal.

Shell (Fig. 19) large, conical, with varying relative height; umbilicus fully covered or more rarely slit-like; individual whorls low and tightly coiled; suture deep; last whorl not expanded; aperture small and low; no umbilicus; protoconch rather large; shell surface irregular with occasional lightly coloured ribs; basic colour pale grey, usually with four well-developed dark brown bands of which the middle pair is much thicker, but bands sometimes only faint; aperture margins brown, about as dark as the bands. Animal (Fig. 20) brown including mantle margins, rarely pale brown.

##### Distribution and habitat.

**Figure 21.**
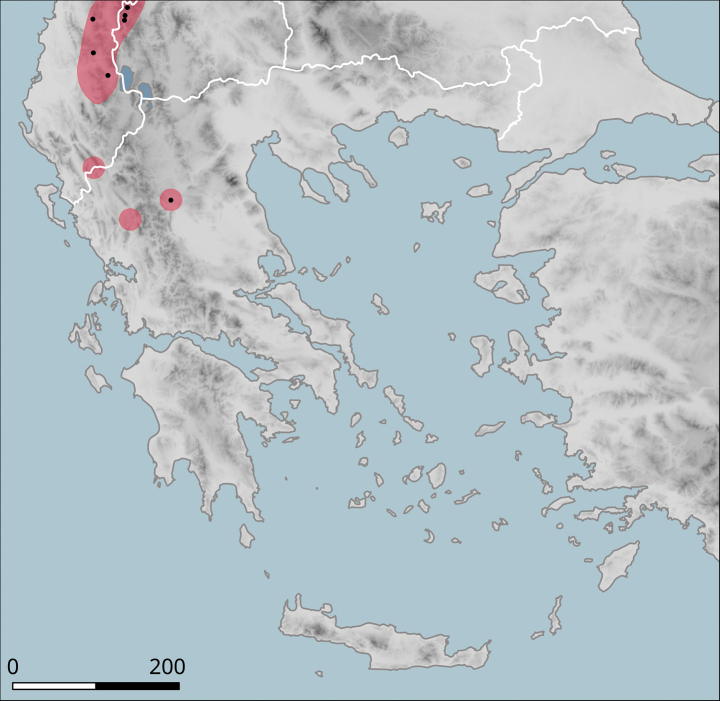
Approximate distribution of Helix (Helix) straminea in Greece. Black dots denote populations sampled for molecular analyses. The western area of occurrence in Greece still requires confirmation.

*Helixstraminea* occurs in the northwest of North Macedonia and in central Albania (Fig. 21); besides that, it is widely distributed in the Apennines (Petraccioli et al. 2021; Korábek et al. 2022). This species is reported here from Greece for the first time; it was found in a floodplain of a small stream north of Kalambaka (Thessaly, central Greece). Two live individuals and several empty shells were collected in a gallery forest dominated by *Platanus*, but with a rich herb understorey. While the mitochondrial haplotype of these specimens belongs to one of the mitochondrial lineages of *H.schlaeflii*, they can be identified as *H.straminea* based on the more conical shell with a narrower last whorl and a smaller aperture (cf. Korábek et al. 2014: fig. 4B). A closely related *H.schlaeflii* haplotype was already recorded in *H.straminea* before in central Albania, where two other sequenced individuals from the same population had *H.straminea* haplotypes as expected (Korábek et al. 2022). However, that population originated from central Albania and all the *H.schlaeflii* haplotypes related to the haplotype from Greece obtained here also originate from central Albania. Although *H.straminea* (as *Helixvladika* (Kobelt, 1898)) was reported from an unspecified locality in the valley of Vjosa at the border between Albania and Greece (Dhora and Welter-Schultes 1996), the current record is far from the range of the species and is perhaps explainable by an introduction. However, there may exist a second population in northern Greece on the east bank of the Ioannina Lake, as documented by one convincing and two possible records posted on iNaturalist (https://www.inaturalist.org/observations/180903383, https://www.inaturalist.org/observations/189208381, https://www.inaturalist.org/observations/121853021). Furthermore, the distribution of *H.straminea* in the Balkan part of its range is very scattered, so it is conceivable that it extends naturally also to northern Greece.

*Helixstraminea* was in the Balkans found in different habitats, from beech forests to shrubs. There is no clear species boundary to the closely related *H.vladika* in the north (northern Albania to central Serbia).

#### ﻿ Helix (Helix) thessalica

Taxon classificationAnimaliaStylommatophoraHelicidae

Boettger, 1886

18FD6CC0-2B72-5732-9C61-87FA98C808A1

Figs 22 , 23

##### References.

Korábek et al. 2016a, 2016b, 2020, 2023b; Korábek and Hausdorf 2024.

##### Description.

**Figure 22.**
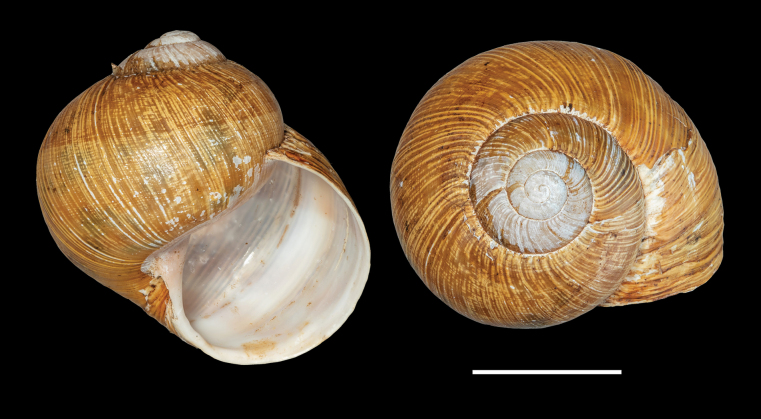
Shell of Helix (Helix) thessalica (Greece, Western Macedonia, between Kastaneri and Livadia; 40.9867°N, 22.3254°E; NMP P6M 42923). Photo R. Coufal. Scale bar: 2 cm.

**Figure 23.**
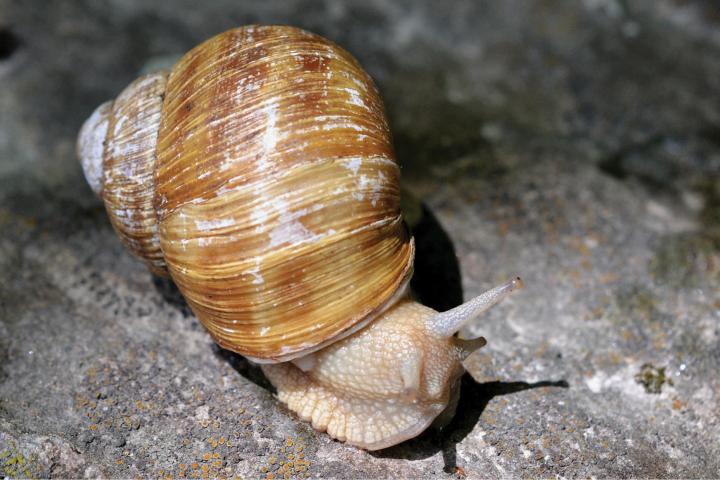
Helix (Helix) thessalica (Greece, Thessaly, Chania; 39.3967°N, 23.0603°E; NMP P6M 30149). Photo O. Korábek.

Shell (Fig. 22) large, globular; body whorl large; very spacious aperture; umbilicus narrow, completely covered or slit-like; shell surface with irregular fine ribs; strongly developed fine spiral grooves (well visible above the aperture); shell covered with a thick periostracum, yellowish brown; bands largely missing in Greek populations; aperture margins white in Greek populations; mantle pale. Animal (Fig. 23) pale brown or yellowish.

##### Distribution and habitat.

**Figure 24.**
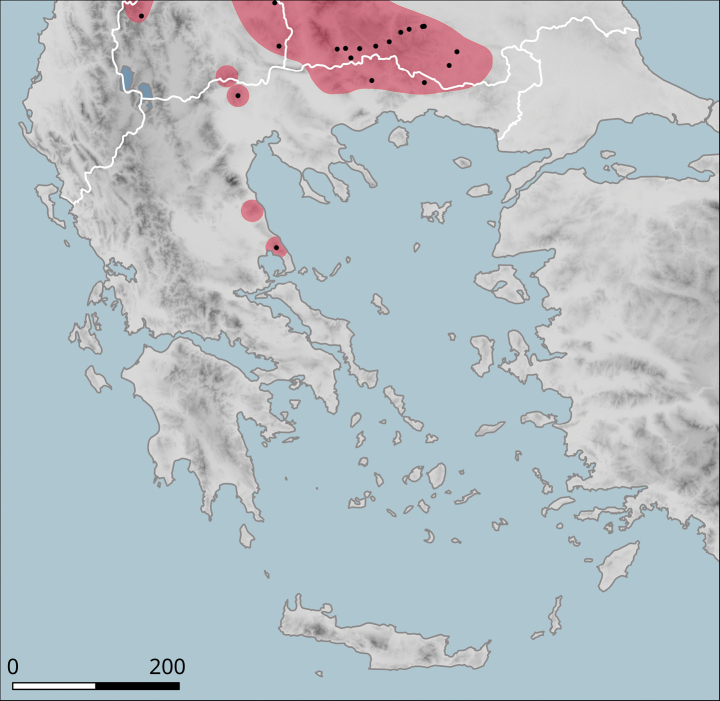
Approximate distribution of Helix (Helix) thessalica in Greece. Black dots denote populations sampled for molecular analyses.

In Greece it lives in higher altitudes. It is relatively broadly distributed in the Rhodopes (Fig. 24), there are isolated occurrences in Thessaly (Ossa/Kissavos, Pelion), and we report it here from the Paiko Mts. (SW of Gevgelija). Its typical habitat in Greece is beech forests, where it can be found in places enriched in nutrients (e.g. along streams, in nettles). Geophilous, but juveniles climb on herbs and can be often found on their leaves.

##### Remarks.

The most similar species in Greece is *H.schlaeflii*. In *Helixthessalica*, the shell is in most cases much more darkly coloured with a distinctly developed periostracum. Greek populations lack any brown colour on the columella, which in turn is characteristic for *H.schlaeflii*. *Helixthessalica* has a dark grey penis, epiphallus and vagina. The description as provided fits the Greek populations, but in other parts of the range the species may be distinctively banded, with brown apertural margins and darker foot.

#### ﻿ Helix (Helix) philibinensis

Taxon classificationAnimaliaStylommatophoraHelicidae

Rossmässler, 1839

5F606D81-DAF0-5B4C-BD92-444959C5BC59

Figs 25 , 26

##### References.

Neubert 2014; Korábek et al. 2022.

##### Description.

**Figure 25.**
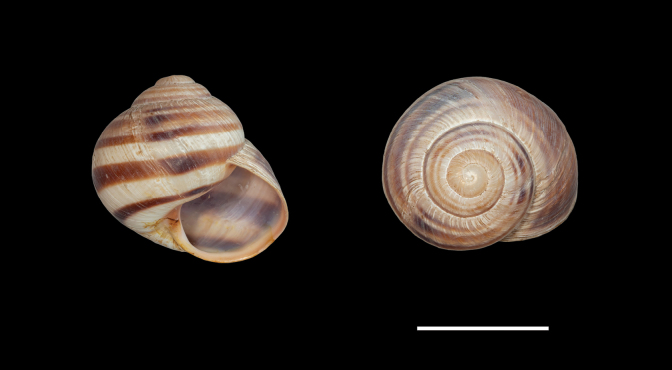
Shell of Helix (Helix) philibinensis (Greece, Western Macedonia, Servia; 40.1788°N, 21.9969°E; NMP P6M 42938). Photo R. Coufal. Scale bar: 2 cm.

**Figure 26.**
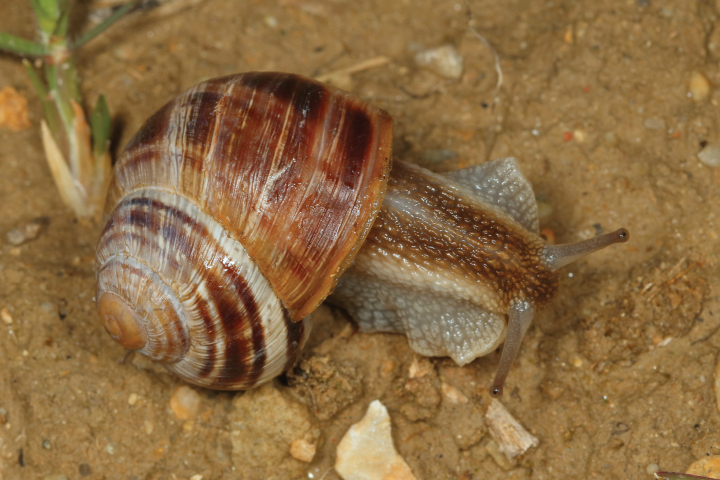
Helix (Helix) philibinensis (Greece, Western Macedonia, Imera; 40.3008°N, 22.0501°E). Photo R. Coufal.

Shell (Fig. 25) small, globular or slightly conical; body whorl expanded but bent downwards, so the aperture is relatively small; no umbilicus; protoconch middle-sized, but relatively large to shell size, making the apex blunt; shell surface with some growth lines but quite smooth; shell colour is whitish to pale brown; bands variously developed, brown on brown shells and with red or purple hue on whitish shells; all bands often separate on older whorls but may fuse towards the aperture; aperture margins straight, usually brown to some degree at least at the columella which may be very dark. Foot (Fig. 26) pale grey with dark brown back.

##### Distribution and habitat.

**Figure 27.**
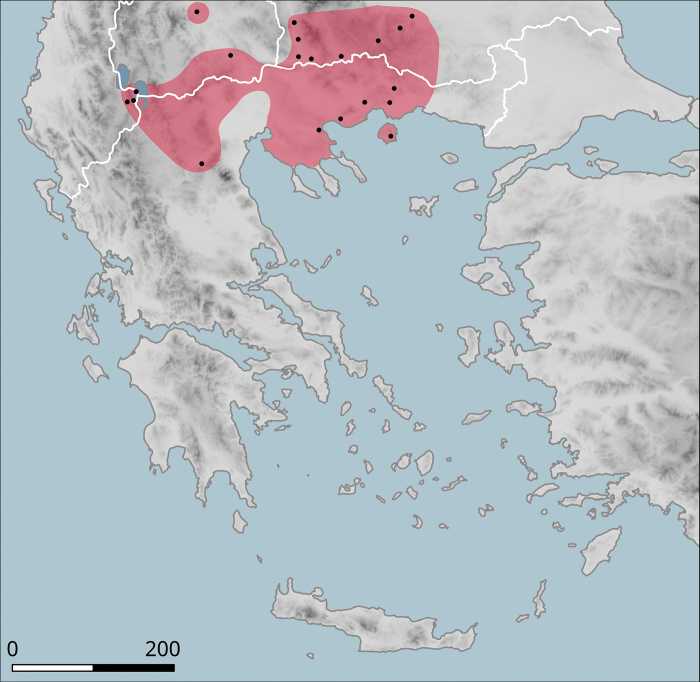
Approximate distribution of Helix (Helix) philibinensis. Black dots denote populations sampled for molecular analyses (including unpublished data).

Distributed from Lake Prespa (northwestern Greece) to the island of Thasos (northeastern Greece) (Fig. 27). The southernmost populations occur on Chalkidiki (the base of the peninsula) and in the south of Western Macedonia (by Servia). In the north, it reaches almost to Skopje in North Macedonia and to Plovdiv in Bulgaria. The core of the range is in Central Macedonia, elsewhere the distribution is highly fragmented. Typically living in open, shrubby habitats, often on rocky slopes, but sometimes found even on forested valley bottoms. It appears to be indifferent to bedrock, as we found it on limestones, but also on granite and gneiss. Though typically resting on the ground, we saw it climbing onto vegetation during rain.

#### ﻿ Helix (Helix) borealis

Taxon classificationAnimaliaStylommatophoraHelicidae

Mousson, 1859

10EF5060-A543-5B47-834E-2B5E80D3F675

Figs 28 , 29

##### References.

Neubert 2014; Korábek et al. 2021.

##### Description.

**Figure 28.**
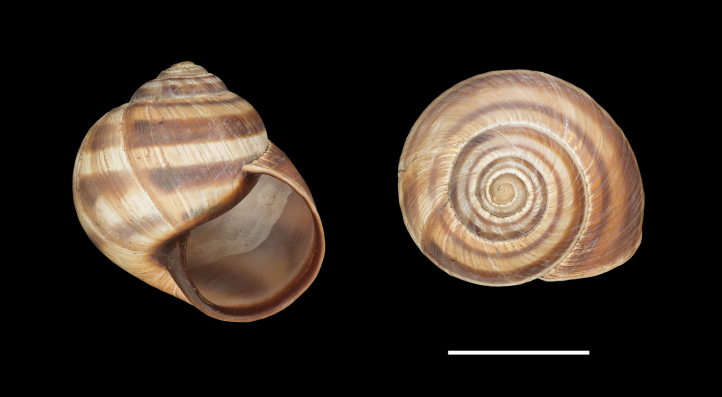
Shell of Helix (Helix) borealis (Greece, West Greece, Zacharo; 37.4973°N, 21.6110°E; NMBE 565401). Photo P. J. Juračka (reproduced from Korábek et al. 2021 with permission). Scale bar: 2 cm.

**Figure 29.**
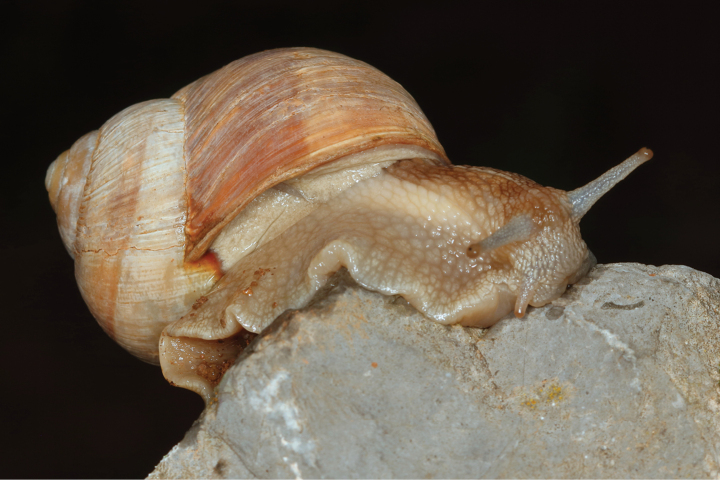
Helix (Helix) borealis (Greece, Thessaly, Loutropigi; 39.1174°N, 22.0413°E). Photo R. Coufal.

Shell (Fig. 28) mid-sized, globular to slightly conical; aperture rounded and in the more globular populations spacious; umbilicus missing; protoconch small; shell surface rather smooth, with fine growth lines; basal colour ranging from nearly white to pale brown; banding pattern varies from completely reduced bands to well-developed and contrasting; the upper three bands may be separated only on the upper whorls or well separated up to the last whorl; aperture margins and the parietal region brown and much darker than the rest of the shell, the colour ranges from vivid pale brown with orange tones (Ionian Islands, Epirus) to dark with purple tones (Peloponnese, Evia); mantle margins pale. Animal (Fig. 29) pale grey or pale brown, often but not always with a dark back of the foot.

##### Distribution and habitat.

**Figure 30.**
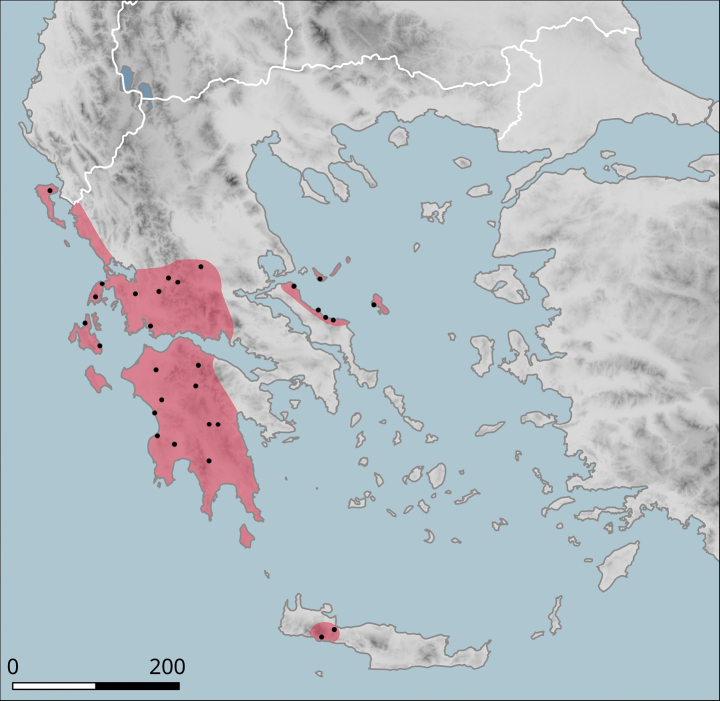
Approximate distribution of Helix (Helix) borealis in Greece. Black dots denote populations sampled for molecular analyses (including unpublished data).

Typical *Helixborealis* is distributed over much of Peloponnese, the east of central Greece (Aetolia, Acarnania, Phocis, Evrytania) and in western Epirus (Fig. 30). It also occurs on the Ionian Islands. The inhabited environments include as disparate types as rocky phrygana, pine forests on sand dunes at the sea level, and open fir forests in the mountains. Geophilous. Besides typical *H.borealis*, there are two other, rather divergent lineages in Greece. One occurs on northern Evia and Northern Sporades. It is found in rocky habitats on limestones. The second has a small range in the mountains of western Crete.

##### Remarks.

Some *H.borealis* populations have a globular shell with shape resembling that of *H.figulina*, but with a smoother, not regularly ribbed surface. There is also always some brown colouration of the apertural margins. In colour, *H.borealis* is often very similar to *H.pelagonesica*, but the two differ in shell shape and in the small protoconch of *H.borealis*. The globular-shelled form of *H.pelagonesica* from the tip of Sithonia (see below) is similar to *H.borealis* also in the shell shape but has a larger protoconch. In Epirus, *H.borealis* occurs syntopically with *H.schlaeflii*. Although the colouration may be similar there, *H.schlaeflii* is much larger and has a large protoconch.

In the mitochondrial phylogeny (Fig. 2), *H.borealis* appears as possibly polyphyletic. However, the relevant parts of the tree are unresolved, so this is likely just an issue of low phylogenetic signal.

#### ﻿ Helix (Helix) lucorum

Taxon classificationAnimaliaStylommatophoraHelicidae

Linnaeus, 1758

57FAD28A-0020-5613-8CAE-28A454E647CE

Figs 31 , 32

##### References.

ICZN 2002; Neubert 2014; Korábek et al. 2018, 2023a; Korábek 2020.

##### Description.

**Figure 31.**
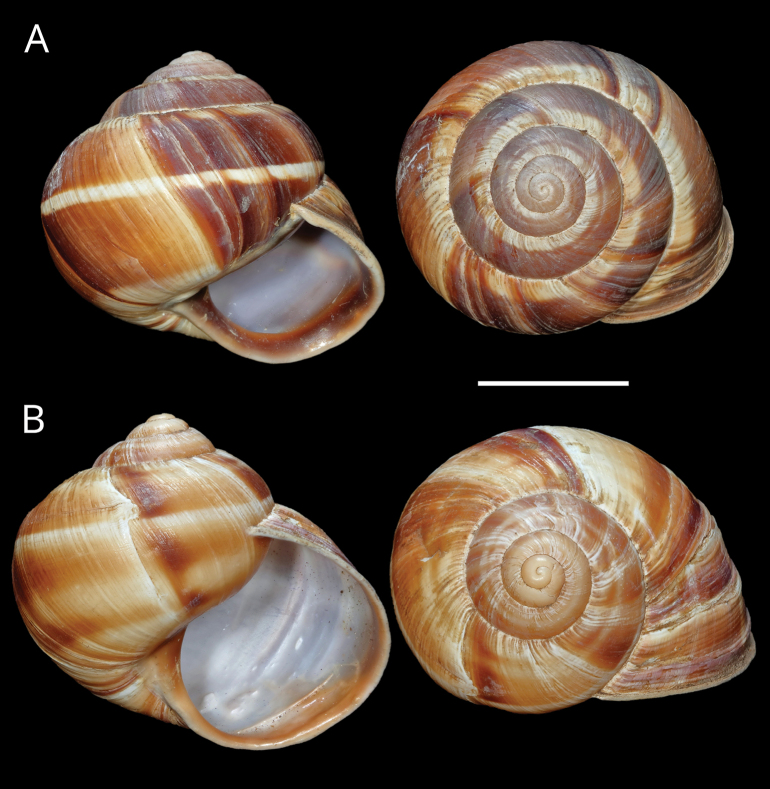
Shells of Helix (Helix) lucorum. A. Greece, Central Macedonia, Stavros; 40.6678°N, 23.6483°E; NMP P6M 30026; B. Türkiye, Manisa Province, Spil Dağı; 38.5822°N, 27.4269°E; NMP P6M 30055. Photo R. Coufal. Scale bar: 2 cm.

**Figure 32.**
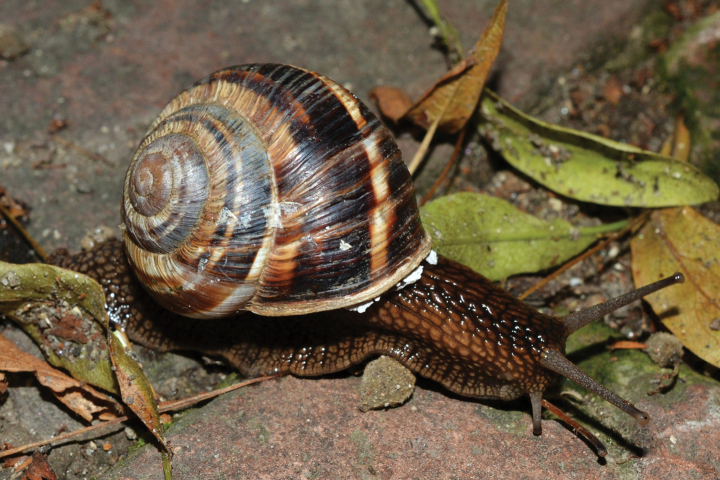
Helix (Helix) lucorum (Czechia, Praha, U Nákladového nádraží; 50.0832°N, 14.4750°E). Photo O. Korábek.

Shell (Fig. 31) mid-sized to large, broadly conical to depressed conical, with relatively narrow body whorl and low aperture; umbilicus present in juveniles but usually fully closed in adults; protoconch small relative to shell size; shell surface smooth; basal colour of the shell whitish, but usually largely covered by dark brown fused bands; characteristic are transverse dark bands marking growth interruptions; conspicuous whitish band along the shell periphery; aperture margins straight at the upper insertion but becoming reflected towards the lower lip and columella, brown inside; columellar margin oblique and often with an internal ridge. Animal (Fig. 32) uniformly brown including the mantle margins; diaphragm paper-like.

##### Distribution and habitat.

**Figure 33.**
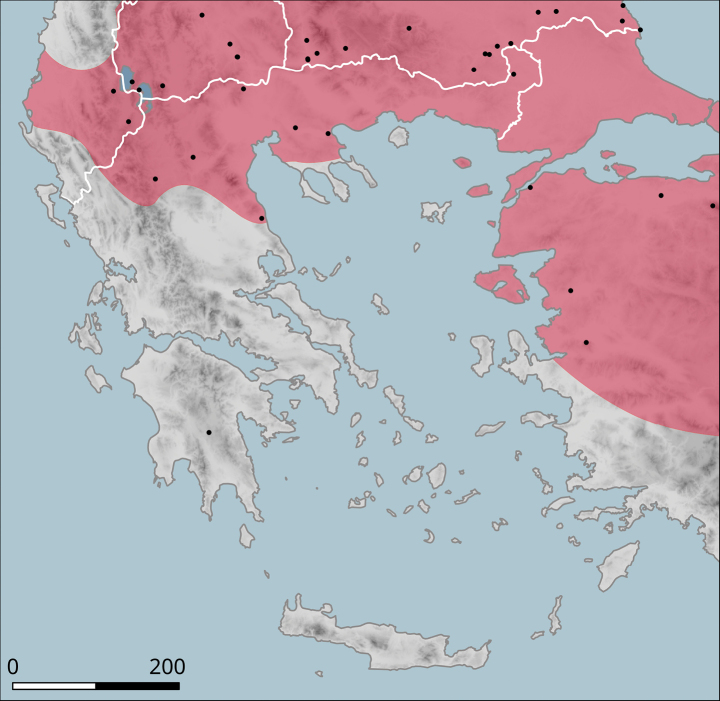
Approximate distribution of Helix (Helix) lucorum in Greece. Black dots denote populations sampled for molecular analyses. Isolated synanthropic occurrences were omitted.

Found commonly in northern Greece except for west of the Pindos Mts. (Fig. 33) but may also be found synanthropically elsewhere (e.g. Peloponnese). Broadly distributed and somewhat invasive species, currently extending its range in Europe. In the northeastern Aegean (Samothraki, Lesvos) a different morphotype with globular shells occurs. *Helixlucorum* lives in various shrubs and herbs and in deciduous forests, but avoids dry Mediterranean types of habitats (phrygana, maquis, exposed rocks). Commonly synanthropic, in some areas exclusively so. Often climbs on vegetation.

##### Remarks.

*Helixlucorum* is very variably coloured and also details of the shell and aperture shape vary. The forms present in mainland Greece are typically very darkly coloured, with a white band on the periphery. The animal is also rather darkly coloured. It has a similar shell shape as *H.straminea* and *H.pelagonesica*, but besides the colour it differs in smooth shell surface and presence of strong transverse banding, visible at least at the bottom of the shell. We found *H.lucorum* syntopic with *H.figulina*, *H.thessalica* and *H.philibinensis*.

#### Subgenus﻿ Pelasga

Taxon classificationAnimaliaStylommatophoraHelicidae

Hesse, 1908

99A7F357-DC73-5A90-950F-B4CFFD2CA00C

##### References.

Hesse 1908; Neubert 2014; Korábek and Hausdorf 2023.

##### Type species.

Helix (Helicogena) pelasgica Kobelt 1904 = *Helixfigulina* Rossmässler, 1839, by subsequent designation (Hesse 1918: 38).

#### ﻿ Helix (Pelasga) figulina

Taxon classificationAnimaliaStylommatophoraHelicidae

Rossmässler, 1839

B9F5F964-764C-536E-944A-C994E8C54847

Figs 34 , 35

##### References.

Neubert 2014; Korábek et al. 2022.

##### Description.

**Figure 34.**
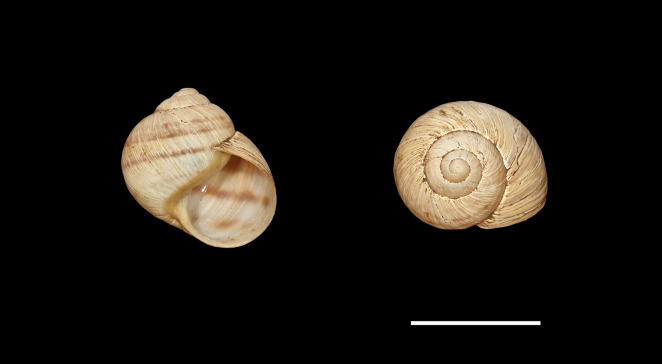
Shell of Helix (Pelasga) figulina (Greece, Western Macedonia, near Kozani; 40.3414°N, 21.8138°E; NMP P6M 42935). Photo R. Coufal. Scale bar: 2 cm.

**Figure 35.**
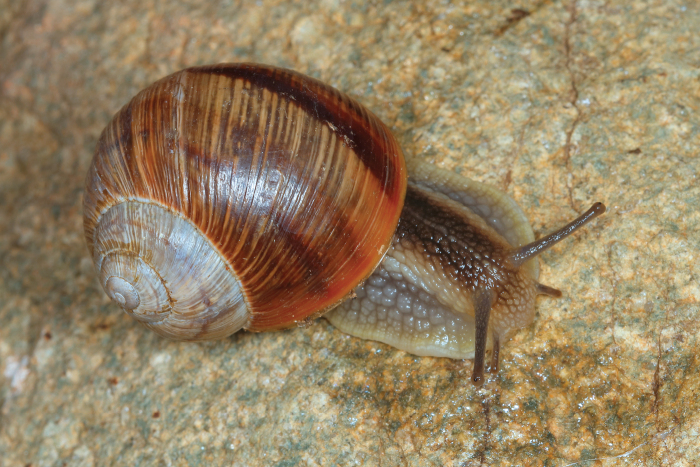
Helix (Pelasga) figulina (Greece, Central Macedonia, Mandres; 40.8681°N, 22.9011°E). A subadult individual lacking a peristome and with a brown periostracum that is already lost on older whorls and is missing in adults. Photo R. Coufal.

Shell (Fig. 34) very small (the smallest *Helix* species in Greece), globular, with large body whorl and spacious aperture; no umbilicus; protoconch very small; shell surface with regular, rounded ribs and lacking spiral sculpture; shell pale greyish or brownish, with the lower two bands narrow and the upper three faint and most often partly fused; aperture margins straight and white; white columella rounded and smoothly transitioning into the palatal area. Animal (Fig. 35) pale brownish with darker, brown or reddish-brown, back, mantle margins pale grey; calcareous diaphragm conspicuously convex and attached to the very margins of the aperture.

##### Distribution and habitat.

**Figure 36.**
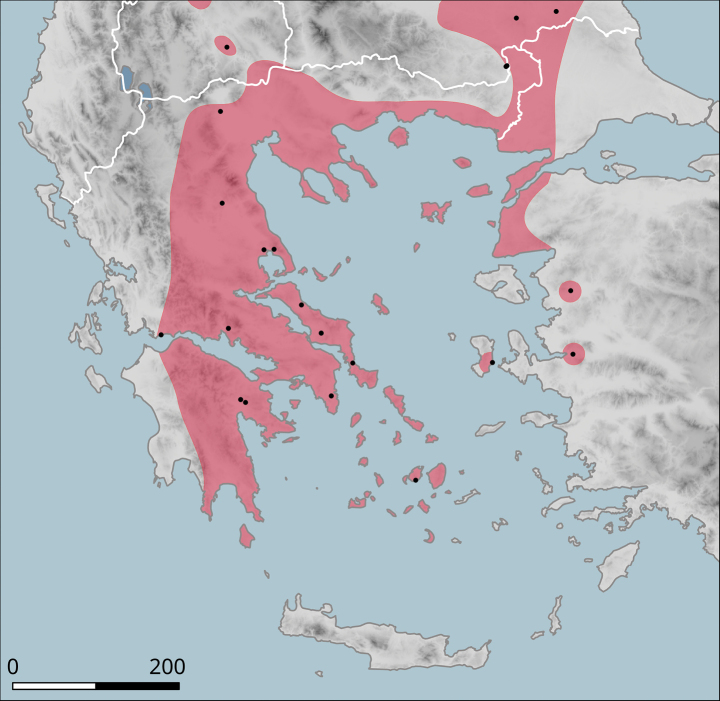
Approximate distribution of Helix (Pelasga) figulina in Greece. Black dots denote populations sampled for molecular analyses (including unpublished data).

Very common species distributed over large part of mainland Greece and the Peloponnese (Fig. 36), but completely missing from the west (<21.5–22.0°E). It is broadly distributed from the southeast of North Macedonia (valleys of Strumica and Vardar, isolated occurrences reported up to Kumanovo) and southeastern Bulgaria (Thrace) to the Aegean islands (Cyclades, Northern Sporades, Lesvos, Samothraki, etc.). It also lives in a small area of western Anatolia (e.g. the ancient Pergamon and Troy). Fossils were found on Crete (Kotsakiozi et al. 2012). It lives in open, often exposed habitats with low vegetation. May be difficult to find alive when inactive because it buries itself into the soil. Geophilous.

##### Remarks.

*Helixfigulina* is easily recognisable due to small size, a very small protoconch, globular shell shape with large aperture, rounded columella smoothly transitioning to the bottom of the previous whorl, completely white aperture margins, and regularly ribbed surface. It may be found syntopically or nearly so with *H.lucorum*, *H.borealis*, *H.philibinensis* and *H.pelagonesica*. *Helixphilibinensis* is the most similar species overlapping in size, but it has a blunter apex, smoother shell surface, smaller aperture which usually has at least partially coloured margins.

### ﻿Other Greek *Helix* species

Several other *Helix* species occur on the islands in the Aegean Sea; we provide here only a brief overview. Majority of them appears to be non-native, indicating a substantial transport of these edible snails in the past.

An endemic, divergent lineage of *H.borealis* lives in western Crete (sample BR21A in Fig. 2). Mitochondrial data group it together with a *H.borealis* lineage endemic to a small area in southwestern Anatolia (Korábek et al. 2021; samples BR1 and BR1C in Fig. 2); multilocus data are not available. Fossil shells of snails of *H.borealis* were also found on Gavdos south of Crete (Welter-Schultes 1998) and on a small islet Astakida northeast of Crete (Mylonas and Vardinoyannis 2022). The Cretan snails have pale brown shells with weakly developed banding and brown aperture margins.

Helix (Helix) pronuba Westerlund, 1879 is more widely distributed in the south of Crete. It is a species with dark brown apertural margins. Its small shells have a granulated upper surface and are often conspicuously banded. The shell is rounded, with a large last whorl and relatively spacious aperture. Besides Crete, *H.pronuba* was also reported from Chalki (Neubert 2014) and Anafi (Psonis et al. 2015), but it is not native to Greece and originates from northern Africa.

The taxonomically problematic group of Helix (Helix) cincta is represented on eastern Aegean islands by a lineage known as *Helixvalentini* Kobelt, 1891 as well as by typical *Helixcincta* Müller, 1774. The group originates from northern Levant (Korábek et al. 2021) and the snails also have brown apertural margins. The former lineage has large, conical shells, and is (or was) found on Kos, Kalymnos, Pserimos and surrounding islets (Neubert 2014; Psonis et al. 2015). Recently, shells were reported from Syrna (Mylonas and Vardinoyannis 2022). Observations posted on iNaturalist (accessed in June 2024) indicate that its main distribution area is in western Syria (governorates Latakia, Tartus). Typical *H.cincta* has a more rounded shell and colouration typically lacking a pattern of fine whitish spots over the bands. The typical form (or its mitochondrial lineage) is distributed on Lipsi, Fournoi, Ikaria, Samos, Chios, and Lesvos.

Helix (Helix) fathallae Nägele, 1901 lives in Greece only on Rhodos. It is most similar in shell shape and colour to *H.philibinensis*, having a larger protoconch and darker apertural margins than *H.cincta*. Furthermore, the columellar triangle is differently shaped, not depressed as in *H.cincta*. It is also a non-native species with problematic taxonomy, but the sample from Rhodos analysed by Korábek et al. (2021) was very closely related to a sample from the type locality. Maroulis et al. (2024) recently examined the genital system and identified the samples from Rhodes as *H.cincta*; we reject this conclusion based on our knowledge of the conchological variation of both taxa as well as the mitochondrial phylogeny.

Helix (Helix) asemnis Bourguignat, 1860 is a middle-sized to large species with whitish shells. Banding pattern varies, but the brown or reddish bands are often not very dark and the three upper bands typically fuse. Aperture margins are purely white and the foot is greyish pink to purely pink in adults. It lives on the islands of Megisti (Mylonas et al. 2019) and Symi (Maroulis et al. 2024). It was reported in the past from Chios and Samos (von Martens 1889), which are detached from its continuous range in southern Anatolia.

Southeastern Aegean islands are inhabited by Helix (Pelasga) nucula Mousson, 1854, whose distribution limits are marked by Lesvos in the north and Kasos in the west (Neubert 2014; Korábek et al. 2022). It is a small species very similar to *H.figulina*, but with dense spiral grooves making the surface of the upper half of the shell granulated. *Helixnucula* is not native to the Aegean and originates from the northern Levant (Korábek et al. 2022).

**Figure 37.**
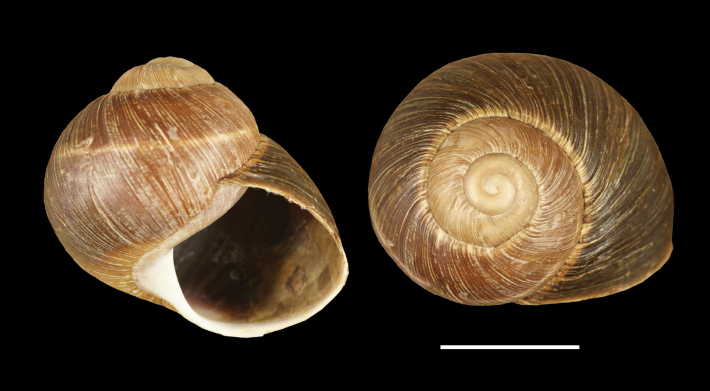
Shells of Helix (Aegaeohelix) godetiana (Greece, Cyclades, Amorgos; ZMB). Photo O. Korábek. Scale bar: 2 cm.

Helix (Aegaeohelix) godetiana Kobelt, 1878 (Fig. 37) is a phylogenetically isolated species of *Helix* that, as findings of subfossil shells of unknown age show, was broadly distributed on the Cyclades and in Dodecanese (Frank 1997; Neubert 2014; Maroulis et al. 2025). However, *H.godetiana* is a declining species and its recent distribution is much smaller (Maroulis et al. 2025). It is extant on Naxos, Amorgos, Astypalaia, Anafi, and Syrna, along with a few small adjacent islets (Gambetta 1929; Mylonas 1985; Neubert 2014; Psonis et al. 2015; Mylonas and Vardinoyannis 2022; Maroulis et al. 2025). Letourneux (1884) collected a live individual on Santorini (Thira). The species is unmistakable: the shell is darkly coloured, with rapidly expanding whorls and large aperture; the embryonal shell is broad.

## ﻿Discussion

Eight of the ~38 currently accepted *Helix* species (MolluscaBase eds 2024) live in continental Greece, which includes species from two (*Helix*, *Pelasga*) of its three subgenera (the third, *Aegaeohelix*, is endemic to the Greek islands) and from all three major clades within the nominotypic subgenus (Korábek et al. 2015). Greece is an important centre of European land snail diversity and the same holds for *Helix*, therefore it is in Greece where we may expect to find narrowly distributed endemic lineages the most.

When revising the eastern distribution limits of *H.schlaeflii*, we discovered two new mitochondrial lineages that form a clade with *H.pelagonesica*. Both have a limited distribution: one is found only on the tip of the Sithonia peninsula, the other lives along a strip of hills that is no more than 50 km in length. The latter lineage is described here as a new subspecies, *Helixpelagonesicathembones*. We decided to introduce a new taxon for four reasons: a) this mitochondrial lineage is distinctive, sister to the lineage found in typical *H.pelagonesica* across its distribution range, b) populations sharing this lineage are characterised by shell shape different from typical *H.pelagonesica* and c) their shell colouration is unique, and d) this lineage and the corresponding morphotype occur in a small area that is geographically isolated from the rest of *H.pelagonesica*’s distribution range. Only a) and b) applies also for the lineage from the tip of Sithonia. These snails are, except for a different shell shape, very similar to typical *H.pelagonesica*, and there is currently no geographic isolation from *H.pelagonesica*. Although the lineage from the tip of Sithonia is more distantly related to typical *H.pelagonesica* in the mitochondrial tree than is the one described here as a new subspecies, we decided not to formally describe it as a new taxon. In *H.pelagonesicathembones* the difference in appearance is more pronounced and the geographic isolation means that the lineage is evolving independently. In contrast, the mitochondrial lineage from the tip of Sithonia could be a remnant of a previously isolated lineage whose nuclear genome now largely merged back with *H.pelagonesica*. Genome-wide data from a detailed sampling of *H.pelagonesica* would be needed to resolve the issue definitely.

The new subspecies appears to have a very restricted distribution range. We speculate that the species may be limited by geological conditions, occurring basically only in a narrow strip of limestones squeezed between flysch to the west and the Thessalian plain to the east (Apostolidis and Koukis 2013). Under this hypothesis, it can be expected to occur up to the vicinity of Kalambaka. However, in Greece, the link between habitats and the occurrence of *Helix* species is in most cases unclear. On the one hand, there are many places where no *Helix* or only *H.figulina* occurs, suggesting that they are unsuitable for most species. On the other hand, it is apparent that in most cases the species live in a variety of habitats. *Helixphilibinensis*, a species with a somewhat scattered distribution, was found by us both on limestones and acidic rocks and on open, exposed, rocky slopes as well as in shaded habitats. *Helixschlaeflii* and *H.borealis* are distributed from phrygana in the low altitudes to altitudes over 1000 m with completely different vegetation and substantially cooler climate. So what is limiting the distribution of Greek *Helix* species is currently unclear. It is possible that interspecific interactions played some role in setting the distributions as especially related species do not co-occur. While H. (Pelasga) figulina lives syntopically with several Helix (Helix) species, syntopy between species of the nominotypic subgenus is in continental Greece limited to a few combinations of species that are phylogenetically distant: *H.lucorum* with *H.philibinensis* or *H.thessalica*, *H.borealis* with *H.schlaeflii*. However, we stress that the biology of the Greek species is generally unknown.

## Supplementary Material

XML Treatment for Helicidae

XML Treatment for Helicinae

XML Treatment for Helicini

XML Treatment for Helix

XML Treatment for Helix

XML Treatment for Helix (Helix) pelagonesicapelagonesica

XML Treatment for Helix (Helix) pelagonesicathembones

XML Treatment for Helix (Helix) schlaeflii

XML Treatment for Helix (Helix) straminea

XML Treatment for Helix (Helix) thessalica

XML Treatment for Helix (Helix) philibinensis

XML Treatment for Helix (Helix) borealis

XML Treatment for Helix (Helix) lucorum

XML Treatment for Pelasga

XML Treatment for Helix (Pelasga) figulina

## ﻿Additional information

### Use of AI

No use of AI was reported.

### Author ORCIDs

Ondřej Korábek https://orcid.org/0000-0002-2522-9165

Petr Dolejš https://orcid.org/0000-0001-9360-7247

Radovan Coufal https://orcid.org/0000-0002-5870-5041

Lucie Juřičková https://orcid.org/0000-0001-5163-1222

Kateřina Kubíková https://orcid.org/0009-0003-7328-0146

Bernhard Hausdorf https://orcid.org/0000-0002-1604-1689

### Data availability

Nucleotide sequences were deposited in the NCBI Nucleotide database; accession numbers are listed in the Supplementary Information.

## Supplementary materials

Supplementary material 1 List of samples used in the phylogenetic analysis with locality and voucher information and GenBank accession numbers Data type ods https://binary.pensoft.net/file/1396808This dataset is made available under the Open Database License (http://opendatacommons.org/licenses/odbl/1.0/). The Open Database License (ODbL) is a license agreement intended to allow users to freely share, modify, and use this Dataset while maintaining this same freedom for others, provided that the original source and author(s) are credited.Ondřej Korábek, Petr Dolejš, Radovan Coufal, Lucie Juřičková, Kateřina Kubíková, Bernhard Hausdorf
